# Development of an endogenous promoter-driven CRISPR/Cas9 system for genome editing in *Fraxinus mandshurica*

**DOI:** 10.48130/forres-0025-0016

**Published:** 2025-08-04

**Authors:** Shangzhu Gao, Mengfan Zhao, Siyu Sun, Xin Fan, Jialin Yan, Ying Xin, Yaguang Zhan, Fansuo Zeng

**Affiliations:** 1 State Key Laboratory of Tree Genetics and Breeding, Northeast Forestry University, Harbin 150040, China; 2 College of Life Science, Northeast Forestry University, Harbin 150040, China; 3 School of Forestry, Northeast Forestry University, Harbin 150040, China

**Keywords:** CRISPR/Cas9, *Fraxinus mandshurica*, *U6* promoter, *FmECP* promoter, *FmPDS*

## Abstract

CRISPR/Cas9-mediated genome editing has revolutionized tree improvement by enabling precise trait modification, accelerating breeding cycles, and enhancing forestry sustainability. *Fraxinus mandshurica*, valued for its desirable traits and adaptability, serves as a strategic focus for the National Reserve Forest Project (NRFP) and forestry germplasm resource breeding and quality improvement in China. Developing a species-specific genome editing system is crucial for valuable yet recalcitrant species like *F. mandshurica*. In this study, the development of a species-specific CRISPR/Cas9 platform is presented for *F. mandshurica*, which incorporates endogenous promoter engineering, sgRNA optimization, light quality modulation, and temperature control protocols to enhance genome editing efficiency. Truncated endogenous *FmU6* promoter variants (*FmU6-6-4* and *FmU6-7-4*) drove sgRNA expression at levels 3.36 and 3.11 times higher than that of the *AtU6-26* promoter. The expression of Cas9 was controlled by the endogenous constitutive *FmECP3* promoter, exhibiting an activity 5.48 times greater than the positive control. A highly active sgRNA4 targeting *FmPDS1/2* was identified, demonstrating a cleavage efficiency of 36.10%. Heat treatment at 37 °C effectively increased the Cas9 cleavage efficiency to 7.77 times that observed at 22 °C. Chimeric albino mutants with an editing efficiency of 18.2% were obtained through transient and stable transformations, combined with light quality optimization and heat treatment during different regeneration stages. The mutation types included nucleotide insertions, deletions, and substitutions, leading to early termination codons and truncated FmPDS1/2 protein. Additionally, mutations in *FmPDS1/2* resulted in albino phenotypes and a reduction in chlorophyll content to 46.44%−58.88%. This optimized system provides a robust platform for functional genomics studies and trait improvement in *F. mandshurica*, with potential applications in forestry biotechnology.

## Introduction

*Fraxinus mandshurica* is a valuable deciduous woody species belonging to the *Oleaceae* family. It is extensively utilized in landscaping, bioenergy, and various industries due to its exceptional resistance and remarkable wood quality^[1]^. *F. mandshurica* serves as the primary afforestation tree species for the National Reserve Forest Project (NRFP) and is a strategic focus for forestry germplasm resources breeding and quality improvement in China, owing to its desirable traits and adaptability^[2]^. However, with the escalating global population, drastic climate changes, and environmental pollution, the demand for timber is projected to increase by 49% by 2050, surpassing the capacity to meet both economic development needs and ecological sustainability^[3,4]^. Consequently, the emphasis on genetic breeding in *F. mandshurica* has shifted towards the development and application of tree varieties that exhibit high yield, superior quality, and enhanced adaptability. The extended growth period, large and complex genome, high genetic heterozygosity, and intricate regeneration capacity present significant challenges to traditional forestry breeding methods, including chemically or radiation-induced mutagenesis and cross-breeding techniques^[5]^. Molecular design offers a promising pathway for the precise modification of specific traits and the efficient creation of new plant varieties.

Currently, CRISPR is recognized as the genome editing technology with the most widely acknowledged developmental potential. The CRISPR/Cas9 system was first utilized in the model plants *Arabidopsis* and tobacco, marking a significant milestone in genetic engineering^[6]^. Since then, it has found extensive applications in genome editing across various crops, such as rice^[7]^, wheat^[8]^, and maize^[9]^, as well as in horticultural crops like tomato^[10]^, lettuce^[11]^, and groundnut^[12]^. Furthermore, its utility has extended to floricultural species, including chrysanthemum^[13]^, and medicinal plants such as patchouli^[14]^. Notably, the application of CRISPR/Cas9 in woody species promotes the creation of novel varieties and enhances timber sustainability through comprehensive studies on timber properties, stress resistance, growth and developmental processes, flower development, and secondary metabolism^[15]^. Taking poplar as an example, multiple CRISPR editing targeting lignin synthesis genes achieves a combinatorial improvement in lignin composition and wood properties, increasing the wood carbohydrate-to-lignin ratio to 228% of that in WT^[16]^. By knocking out *4CL1*, the lignin content in poplar wood is effectively reduced by 12.8%, resulting in a tensile strength of 313.6 ± 6.4 MPa^[17]^. CRISPR-based genome editing of *PtrLBD39/22* leads to a decrease in cellulose content and an increase in lignin content, severely inhibiting the formation of tension wood (TW)^[18]^. CRISPR-mediated knockout mutants of *CESA4* exhibit developmental retardation, physiological abnormalities, altered cell wall structure, and improved saccharification efficiency^[19]^. These remarkable advances facilitate fundamental research, tree breeding, and germplasm improvement programs at an accelerated pace^[16]^.

The CRISPR/Cas9 system consists of two primary components: the Cas9 nuclease, which cleaves DNA, and the single guide RNA (sgRNA) that directs this action^[20]^. For effective genome editing in plants, it is essential to utilize appropriate promoters that enable the expression of both sgRNA and Cas9. The sgRNA serves as a small RNA with a specific secondary structure, typically synthesized by RNA polymerase III (pol III) promoters, such as *U3* and *U6*^[21]^. Notably, the *U3* promoter is predominantly employed in the genome editing of monocotyledonous plants, while the *U6* promoter is commonly utilized in dicotyledonous plants^[22]^. The expression of Cas9 is generally activated by pol II promoters, including the CaMV35S and *ubiquitin* (*Ubi*) promoters^[22,23]^. Various studies have demonstrated that the application of species-specific promoters significantly enhances the expression levels of sgRNA and Cas9, representing a robust and efficient CRISPR/Cas9 system. To date, genome editing systems have been successfully established and optimized in maize^[24]^, white birch^[25]^, soybean^[26]^, cotton^[27]^, grape^[28]^, pigeonpea^[29]^, banana^[30]^, and various other species by leveraging endogenous *U6* promoters to drive sgRNA expression. The same *U6* promoter exhibits varying transcriptional activities across different species, which may be attributed to differences in transcription, translation, and regulatory mechanisms among these species^[26]^. For instance, the *AtU*6 promoter effectively mediates sgRNA expression in tobacco, tomato, and poplar but exhibits low efficiency in wheat and rice^[31]^. Compared with *AtU6-26*, *MtU6-5*, and *MtU6-6* promoters, the endogenous *MsU6* promoter from alfalfa effectively enhances sgRNA expression and enables simultaneous editing of multiple genes in *Medicago sativa*^[32]^. The endogenous *GhU6.3.3* promoter drives sgRNA expression at levels six to seven times higher than *AtU6-29*, and the editing efficiency is four to six times higher than that of *AtU6-29* in cotton^[27]^. The genome editing efficiencies of pigeonpea achieve 8.80% *in planta* and 9.16% *in vitro* via the editing vector driven by the *CcU6_7.1* promoter^[29]^. Furthermore, the screening of Cas9 promoters (constitutive, inducible, or tissue-specific) has led to spatiotemporal control and higher efficiency in targeted editing in species such as *Arabidopsis*^[33,34]^, grape^[28]^, maize^[35]^, tomato^[36]^, and citrus^[37]^. Species-specific *VvU6* and *UBQ2* promoters lead to higher editing efficiencies by effectively promoting the expression of sgRNA and Cas9 in grapes^[28]^.

Phytoene desaturase (PDS) catalyzes the conversion of colorless phytoene into colored carotenoids in the carotenoid biosynthesis pathway. Silencing *PDS* gene expression causes plants to exhibit a unique albino phenotype^[38]^. Given that *PDS* gene alterations produce an observable phenotype, it has become a popular target gene in plant genome editing systems. Current research on species such as white birch^[25]^, poplar^[39]^, larch^[40]^, and bamboo^[41]^, has used the *PDS* gene to confirm the establishment and optimization of their CRISPR/Cas9 system. Furthermore, these studies have added to the evidence for the effects of *PDS* gene knockout, such as reduced chlorophyll content, albino phenotypes, and plant growth retardation/dwarfism.

*F. mandshurica*, a valuable timber species, holds significant ecological and economic importance. Genome editing technologies have emerged as transformative tools in forestry research, enabling precise genetic improvements in woody species. However, the genetic enhancement and acquisition of novel germplasm in forest tree species present considerable challenges, primarily due to their prolonged sexual reproduction cycles and the recalcitrant, complex nature of their asexual propagation. In this study, a CRISPR/Cas9-mediated genome editing technique specifically tailored for *F. mandshurica *was developed, addressing the limitations inherent in conventional breeding methods. Several endogenous pol II and pol III promoters with high activity were meticulously identified, and a comprehensive investigation was conducted to elucidate the effects of species-specific promoters, sgRNA sequences, temperature, and light quality on sgRNA and Cas9 activity, as well as genome editing efficiency. By integrating transient transformation methodologies that facilitate rapid gene functional analysis and the generation of T-DNA-free mutants with stable transformation approaches enabling the production of T-DNA-positive mutants, an optimized and efficient pathway for acquiring *F. mandshurica* mutants was established. Consequently, this study provides reliable technical support for gene function studies, targeted mutant construction, and the creation of novel varieties of *F. mandshurica*. Furthermore, this work lays a foundational framework for implementing genome editing technologies in recalcitrant woody species.

## Materials and methods

### Plant materials and culture conditions

The seeds of *F. mandshurica* were taken from the experimental forest of the Northeast Forestry University, sterilized, and grown in Woody Plant Medium (WPM). The poplar and birch seedlings were cultured in WPM. The *Nicotiana tabacum* seedlings were cultured in Murashige and Skoog (MS) medium. All plants were grown at 25 ± 2 °C and 60% relative humidity under a photoperiod of 16 h light/8 h dark.

### Analysis and cloning of endogenous promoters

Based on the conservation of *U6* snRNA (small nuclear RNA) among different species, the sequences of *Arabidopsis* and soybean were used to identify the *U6* snRNA of *F. mandshurica*. The 1.5 kb sequences upstream were identified as *FmU6* promoters from the genome. The promoters were analyzed using PlantCARE and aligned by DNAMAN. According to the number of CAAT-boxes, the promoters were truncated with different sequence lengths.

Eleven highly expressed genes, including non-tissue-specific genes, and *ELONGATION FACTOR-1 alpha* (*EF-1α*) genes, were selected based on transcriptome data of *F. mandshurica*. The heatmaps were shown by TBtools. The 2 kb fragments upstream of these genes were isolated and cloned as endogenous constitutive promoters of *F. mandshurica* (*FmECPs*). Specific primer pairs were designed for promoter cloning (Supplementary Table S1).

### Vectors construction

The pNC-121-pro, in which the 35S promoter of pBI121 was replaced with the NC frame, was used to detect promoter activity of *FmU6*s and *FmECPs*. The *AtU6-26* promoter was cloned into pNC-121-pro as a positive control, while empty vector was used as a negative control. Primers for vector construction were listed in Supplementary Table S2. Recombinant plasmids, pNC-121-proFmU6s and pNC-121-proFmECPs, were used for the transformation of *F. mandshurica*, tobacco, poplar, and birch.

The *FmU6-6-4*/*FmU6-7-4* was inserted into pEgP237-2A-gfp between HindⅢ and BsaI, and the *FmECP3* promoter was inserted into pEgP237-T2A-GFP between AscI and XbaI using the Seamless Assembly cloning kit (C5891, CloneSmarter, USA), which was named as pEgU6E3/pEgU7E3. The oligo DNA for gRNA target was ligated into the optimized CRISPR/Cas9 expression cassette by T4 DNA Ligase (EL0014, Thermo Fisher Scientific, USA). The pEgU6E3/pEgU7E3-FmPDS-sgRNA4/6 constructs were used for *F. mandshurica* transformation.

### Quantitative real-time PCR analysis and selection of target sequences

Protein sequences of PDS in different species were retrieved from NCBI. Multiple sequence alignment was performed by DNAMAN. The target sequences of CRISPR/Cas9 were designed by an online program (http://skl.scau.edu.cn/targetdesign/ and https://crispr.dbcls.jp/), and the efficiency was predicted by www.crispredict.org. The first two target sequences were used to construct genome editing vectors, and primers were listed in Supplementary Table S2. Total RNA was extracted using the CTAB method. Subsequently, reverse transcription was conducted using the PrimeScript™ RT reagent kit with gDNA Eraser (RR047, Takara, China), while qRT-PCR was performed with the TransStart® Top Green qPCR SuperMix (AQ132, TransGen Biotech, China). Specific primer pairs, detailed in Supplementary Table S3, were designed for expression detection. The Cas9 enzyme *in vitro* digestion kit (PC1400, Invogen Tech. Co., China) and the sgRNA transcription kit (PC1380, Invogen Tech. Co., China) were used to determine the cutting efficiency of different gRNA *in vitro*. Primers were listed in Supplementary Table S4. The relative cutting efficiency of different gRNA under different temperatures *in vivo* was performed as previously described^[42]^.

### *Agrobacterium*-mediated transformation of *F. mandshurica*

The GV3101 strain was used for plant genetic transformation. The process of *Agrobacterium*-mediated transformation in *F. mandshurica* according to the previous method^[43]^. After regeneration of adventitious shoots for 7 d, the explants were transferred to selective medium with 50 mg/L Kanamycin (Kan). During the first 14 d of selection, adventitious shoots were treated at 37 °C for 24 h and recovered at 24 °C for 24 h, moving in cycles.

To explore the impact of light quality on tissue culture regeneration, light-emitting diode (LED) lights served as the artificial light, delivering 12 μmol/m^2^/s and 40 W light. The white light emitted a wavelength range of 410–690 nm, while the red and blue lights had wavelength ranges of 600–900 nm (peak at 612 nm) and 410–540 nm (peak at 435 nm), respectively. The explants at different stages of the regeneration process (including adventitious shoot regeneration, adventitious shoot elongation, and rooting) were exposed to various light quality combinations, including white, red light, as well as blue-to-red (B:R) ratios of 1:4 and 2:3.

Transient transformation was conducted following the methodology detailed by Liang et al. with some modifications^[44]^. In brief, the 10-day-old seedlings were subjected to treatment with a hypertonic solution (WPM + 25% sucrose) for 30 min. The *Agrobacterium* was adjusted to an OD_600 _= 0.6 with the solution [WPM + 10 mM CaCl_2 _+ 10 mM MES + 200 µM AS + 200 mg/L DTT + 0.02% (W/V) Tween-20 + 2 mg/L 5-aza], which the whole seedlings were treated with for 4 h using the vacuum infiltration method. The plantlets were moved into WPM solid medium [WPM + 10 mM CaCl_2 _+ 10 mM MES + 100 µM AS + 200 mg/L DTT + 0.02% (W/V) Tween-20 + 2 mg/L 5-aza] for 48 h in darkness. According to different purposes, the seedlings were treated differently: (1) The samples were collected to determine the activity of different promoters, by qRT-PCR and *β*-glucuronidase (GUS) staining with the GUS kit (SL7160, Coolaber, China). (2) Seedlings were transferred to incubators with different temperatures (22, 28, 32, and 37 °C) for 24 h to detect the effect of temperature on cutting efficiency. (3) To obtain transient mutants and preliminary research of gene function, the whole plantlets were planted vertically on selection medium (WPM + 50 mg/L Kan) for 7 d, during which they were treated at 37 °C for 3 h a day.

### DNA extraction, mutation identification, and genotyping analysis

Total DNA was extracted using the CTAB method. The fragments of DNA, including target sequences, were cloned by Phanta Max Super-Fidelity DNA Polymerase (P505, Vazyme Biotech Co., Ltd, Nanjing, China) and sequenced on the Sanger and HI-TOM platform. DSDecodeM and Hi-TOM were used to analyze the sequencing results. Primers for transgenic plants identification and sequencing were listed in Supplementary Table S5. Detection kits (G0613W, Grace Biotechnology, Suzhou, China) were employed for the quantification of photosynthetic pigments, including chlorophyll, chlorophyll a, and chlorophyll b content. SWISS-MODEL software was used to predict tertiary structure.

### Statistical analysis

The data were analyzed using IBM SPSS 22, and the results were expressed as the mean ± standard deviation (SD). Statistical significance was set at *p* < 0.05. All experiments were conducted and analyzed in at least triplicate.

## Results

### Identification and truncation of *U6* promoters in *F. mandshurica*

**Figure 1:**
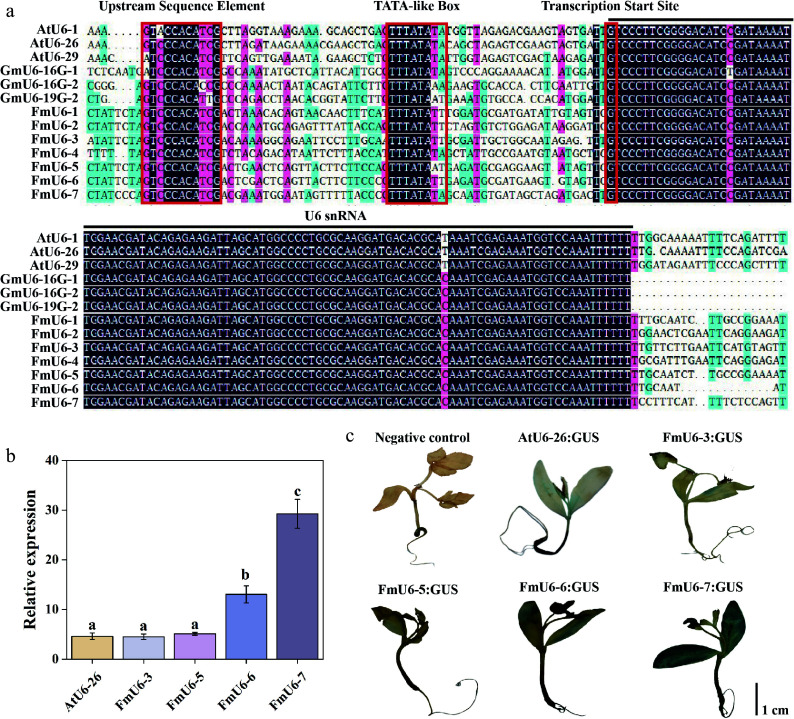
Identification and activity validation of *FmU6* promoters in *F. mandshurica*. (a) Multiple alignments of *F. mandshurica*, *Arabidopsis,* and soybean *U6* gene and promoter sequences. The red boxes with solid lines indicate upstream sequence element (USE), TATA-like box, and the transcription start site. The black line shows the *U6* snRNA transcript. (b) The relative expression levels of *GUS* driven by the promoters of *AtU6-26*, *FmU6-3*, *FmU6-5*, *FmU6-6*, and *FmU6-7* in seedlings of *F. mandshurica*. Values represented mean ± SD (n = 3); different letters indicated significant differences (*p* < 0.05). (c) GUS staining driven by the negative control, promoters of *AtU6-26*, *FmU6-3*, *FmU6-5*, *FmU6-6*, and *FmU6-7* in *F. mandshurica* seedlings. The scale bar represents 1 cm.

In genome editing of dicotyledonous plants, the promoter of endogenous *U6* snRNA is frequently utilized to drive the expression of gRNA. According the conservation of *U6* snRNA sequences across different species, the BLAST search was performed in the *F. mandshurica* genome using *Arabidopsis* *U6* (*AtU6-1*, *AtU6-26*, and *AtU6-29*) sequences as references, and identified seven homologous sequences of 102 bp in *F. mandshurica* (Fig. 1a). All the seven *FmU6* snRNA exhibited high homology with the *U6* snRNA transcription sequences of *Arabidopsis,* and soybean, but significant differences were observed in their promoter regions (Supplementary Fig. S1). However, the examination of the 100 bp sequences located upstream of *FmU6* snRNA revealed that they all contained typical pol III promoter elements: the plant-specific upstream sequence element (USE) situated around –70 bp and a TATA-like box present at positions –28 to –30 bp.

Based on the homology with *AtU6* promoters and the number of transcriptional enhancers (e.g., CAAT boxes) within *FmU6* promoters, four of seven *FmU6* promoter sequences were cloned, namely *FmU6-3*, *FmU6-5*, *FmU6-6*, and *FmU6-7* promoters. To investigate whether these *FmU6* promoters could effectively drive gene expression, the four promoters were individually inserted upstream of the *GUS* gene in the pNC-121-pro vector. All four promoters facilitated *GUS* expression in *F. mandshurica* seedlings, with *FmU6-6* and *FmU6-7* exhibiting higher promotive capabilities than *FmU6-3* and *FmU6-5* (Fig. 1b, c). Specifically, the promoter activities of *FmU6-6* and *FmU6-7* were 2.83-fold and 6.35-fold of the *AtU6-26* promoter, respectively, which may be correlated with the higher number of CAAT boxes in the sequences. Therefore, *FmU6-6* and *FmU6-7* promoters were selected for further investigation.

**Figure 2:**
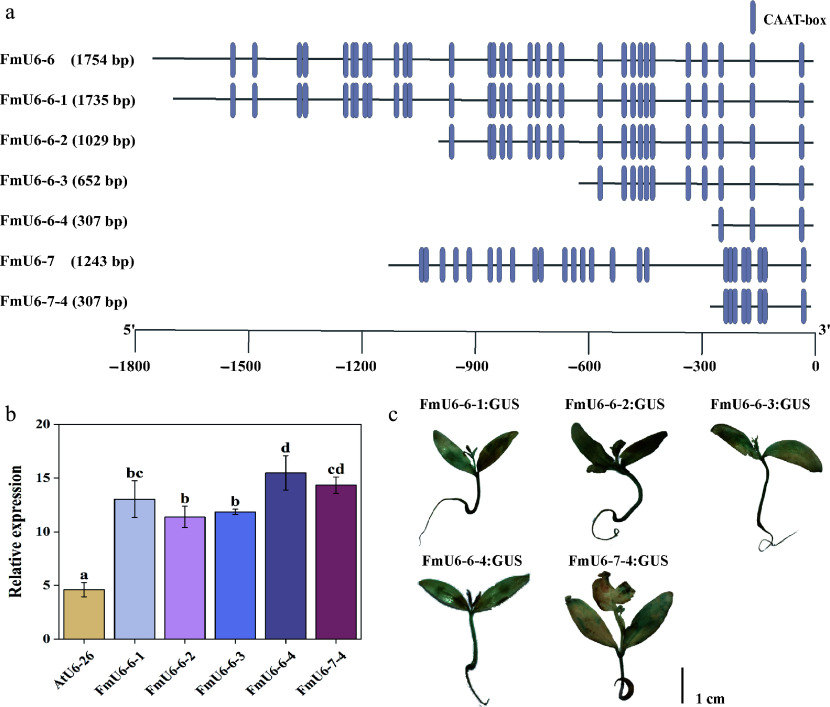
Functional verification of *FmU6* promoter fragments. (a) Truncation of *FmU6-6* and *FmU6-7* promoters. (b) The relative expression levels of *GUS* driven by the promoters of *AtU6-26*, *FmU6-6-1*, *FmU6-6-2*, *FmU6-6-3*, *FmU6-6-4*, and *FmU6-7-4* in seedlings of *F. mandshurica*. Values represented mean ± SD (n = 3); different letters indicated significant differences (*p* < 0.05). (c) GUS staining in *F. mandshurica* seedlings. The scale bar represents 1 cm.

Based on the number of CAAT boxes, the *FmU6-6* and *FmU6-7* promoter sequences were truncated to different lengths, including *FmU6-6-1* (1,735 bp), *FmU6-6-2* (1,029 bp), *FmU6-6-3* (652 bp), *FmU6-6-4* (307 bp), and *FmU6-7-4* (307 bp) (Fig. 2a). *GUS* gene expression analysis and GUS staining indicated that all five truncated fragments of *FmU6* promoter possessed transcriptional activity and significantly exceeded the control levels (Fig. 2b, c). Notably, *FmU6-6-4* and *FmU6-7-4* promoters, truncated to approximately 300 bp, demonstrated the highest promotive activity, which was approximately 3.36 and 3.11 times that of the *AtU6-26* promoter, respectively. Consequently, the shortest fragments, *FmU6-6-4* and *FmU6-7-4* promoters, were utilized to induce the production of gRNA in the CRISPR/Cas9 system of *F. mandshurica*.

### Screening and detection of high-activity endogenous constitutive promoters (ECP) in *F. mandshurica*

**Figure 3:**
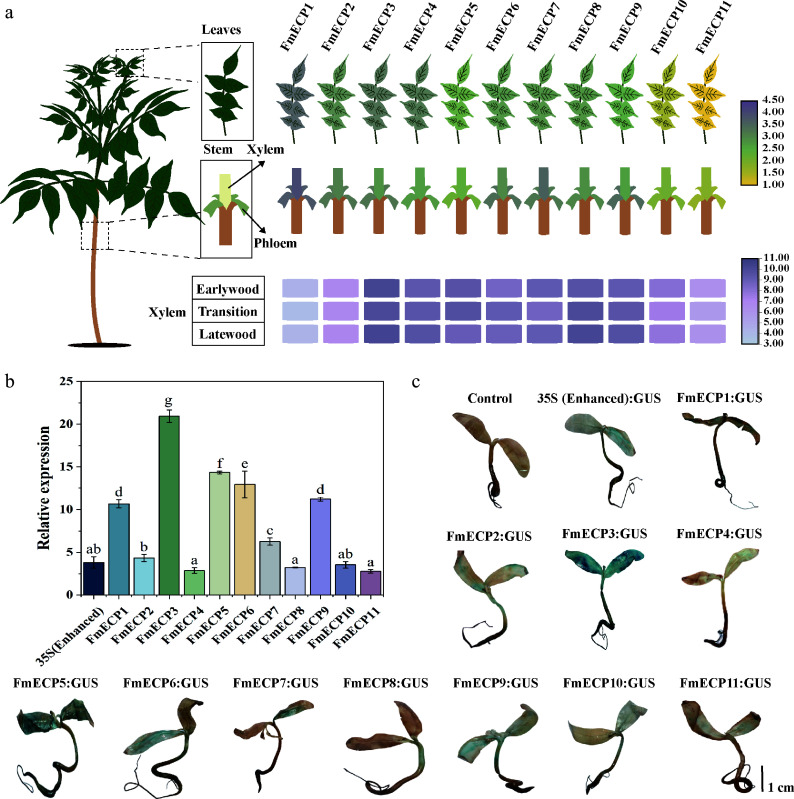
Functional verification of *FmECP* promoters in *F. mandshurica*. (a) The expression profiles of genes driven by *FmECP* promoters, including the relative expression levels in leaves, xylem, phloem, earlywood, transition, and latewood. (b) The relative expression levels of *GUS* driven by 35S (enhanced) and *FmECP* promoters in seedlings of *F. mandshurica*. Values represented mean ± SD (n = 3); different letters indicated significant differences (*p* < 0.05). (c) GUS staining in *F. mandshurica* seedlings. The scale bar represents 1 cm.

The utilization of ECP effectively promotes *Cas9* expression in plants, thereby enhancing genome editing efficiency^[28]^. Based on RNA-seq of various tissues (xylem, phloem, leaf) and developmental stages (earlywood, transition, latewood) in *F. mandshurica*, nine highly expressed genes with non-temporal or non-spatial specificity were observed, and two *EF-1α* genes, the promoters of which were named endogenous constitutive promoter 1−11 (*FmECP1−11*), respectively (Fig. 3a). The promoters were isolated as endogenous promoters to induce *Cas9* expression and assessed their activity in *F. mandshurica* seedlings with the 35S promoter as a control. Four promoters exhibited lower activity than 35S (promoters of *FmECP4*, *FmECP8*, *FmECP10*, *FmECP11*), while six promoters demonstrated significantly higher activity than 35S (promoters of *FmECP1*, *FmECP3*, *FmECP5*, *FmECP6*, *FmECP7*, *FmECP9*) (Fig. 3b). *FmECP3* promoter exhibited the highest activity, 5.48-fold of 35S, followed by promoters of *FmECP5* and *FmECP6*, with activities of 3.76 and 3.39 times of 35S, respectively. Staining results indicated that these three promoters could effectively drive *GUS* expression in different tissues of *F. mandshurica* seedlings (Fig. 3c). The findings demonstrated that the *FmECP3* promoter could serve as an efficient promoter in the genome editing system of *F. mandshurica*.

### Selection of the target site and construction of the CRISPR/Cas9 vector in *F. mandshurica*

**Figure 4:**
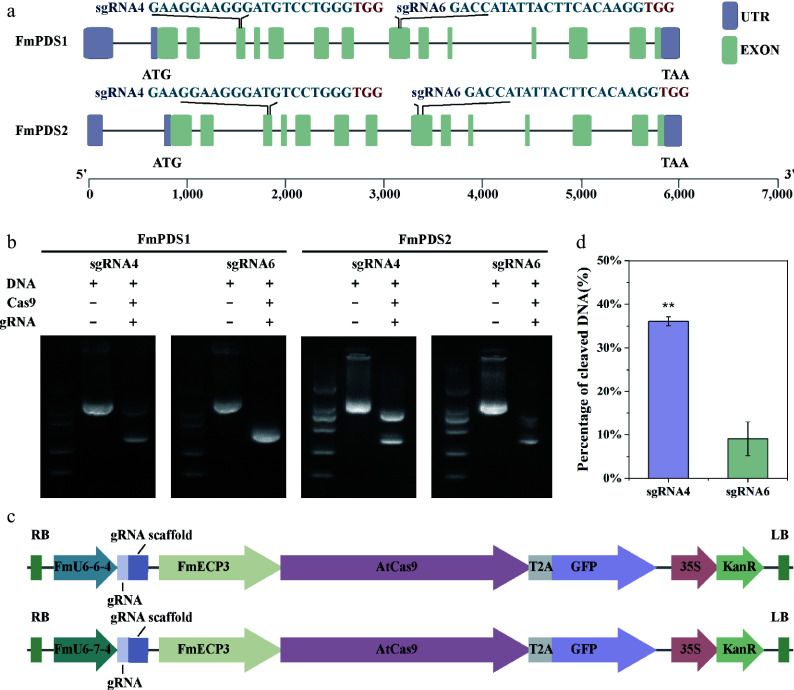
SgRNA targeting sites in *FmPDS1/2* and schematic structure of genome editing vectors. (a) Schematic position of the two gRNAs targeting *FmPDS1/2* genes. The selected target sequences are blue, and the PAM sequences are red. The purple box indicates UTR; the green box represents exon; the grey line represents intron. (b) Determination of the cutting efficiency of different gRNA *in vitro*. (c) The CRISPR/Cas9 vector structure of *F. mandshurica* named pEgU6E3 and pEgU7E3, respectively. (d) Determination of cleaving efficiencies at the different target sites. Asterisks indicate levels of significance (*t*-test; **p < 0.01).

Defects in *PDS* gene function result in the albino phenotype of plants, making *PDS* a convenient gene and indicator in genome editing systems. The *F. mandshurica* genome contains two highly homologous *PDS* genes: Chr16G002539 on chromosome 16 (hereafter named *FmPDS1*) and Chr07G002414 on chromosome 7 (hereafter named *FmPDS2*) (Fig. 4a; Supplementary Fig. S2). The two paralogues have 14 exons separated by 13 introns in each and consist of 6,055 and 6,011 bp of transcript sequence, respectively, sharing 71.37% identity in nucleotide coding sequence. The amino acid sequences of FmPDS1 and FmPDS2, with lengths of 1,734 and 1,740 bp, respectively, shared 92.59% identity at the amino acid level and possessed typical characteristics of plant phytoene desaturases, including the dinucleotide binding motif, putative substrate conserved motif, and carotenoid binding domain (Supplementary Fig. S3). gRNAs were designed for both *FmPDS1* and *FmPDS2* using online tools, in which gRNA4 and gRNA6 designed to target two conserved sites located showed the highest efficiency (Fig. 4a). The sgRNA4 binding to *FmPDS1* and *FmPDS2* was located on the third exons of two genes (red letters in Supplementary Fig. S2); and the sgRNA6 binding to *FmPDS1* and *FmPDS2* was located on the eighth exons of two genes (blue letters in Supplementary Fig. S2). The corresponding sequences were constructed into the pEgU6E3 and pEgU7E3 vectors, in which Cas9 was induced by the *FmECP3* promoter and gRNA4 was driven by *FmU6-6-4* and *FmU6-7-4* promoters, respectively (Fig. 4c). The addition of Cas9 protein and gRNA effectively cleaved DNA *in vitro* (Fig. 4b). Furthermore, the cleavage activity of Cas9 at these two sites was determined *in vivo* through the TCEP method, revealing that the efficiency targeting gRNA4 reached 36.10%, which was 3.97-fold of gRNA6 (Fig. 4d). Therefore, gRNA4 was selected for further research.

### Temperature effect on CRISPR/Cas9-mediated genome editing

**Figure 5:**
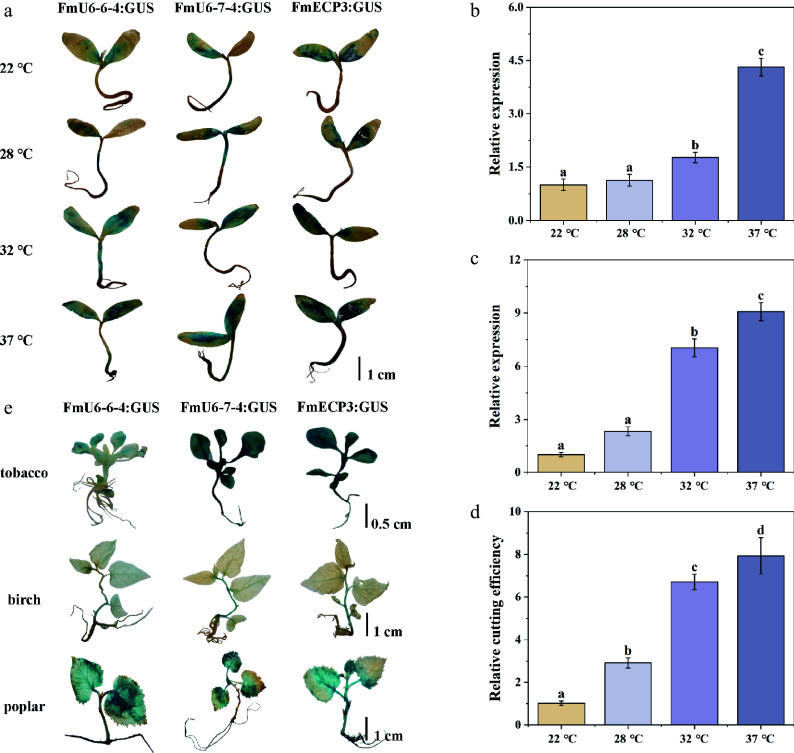
Temperature effect on CRISPR-Cas9-mediated genome editing. (a) The staining of GUS driven by *FmU6-6-4*, *FmU6-7-4*, and *FmECP3* promoters under different temperature (22, 28, 32, 37 °C) treatments in *F. mandshurica* seedlings. The scale bar represents 1 cm. (b) The relative expression level of gRNA driven by *FmU6-7-4* promoter. (c) Cas9 driven by *FmECP3* promoter in seedlings of *F. mandshurica* under different temperature treatments. (d) Determination of the cutting efficiency under different temperature treatments. Values represented mean ± SD (n = 3); different letters indicated significant differences (*p* < 0.05). (e) GUS staining in tobacco, birch, and poplar seedlings. GUS was under the control of promoters of *FmU6-6-4*, *FmU6-7-4*, and *FmECP3*. The scale bar represents 1 cm.

To efficiently and rapidly investigate the impact of temperature on genome editing, transient transformation of *F. mandshurica* seedlings was exposed to various temperature treatments (22, 28, 32, and 37 °C). The results indicated that an increase in temperature effectively enhanced the promoter activities of *FmU6-6-4*, *FmU6-7-4*, and *FmECP3* (Supplementary Fig. S4; Fig. 5a), as well as gRNA and *Cas9* expression levels (Fig. 5b, c). The transcriptional expression levels of gRNA and *Cas9* at 37 °C were 4.32 times and 9.08 times respectively at 22 °C. The relative cleavage efficiency mediated by the pEgU7E3 vector at 37 °C was 7.77-fold of that at 22 °C, demonstrating the steady increase in cleavage efficiency with temperature (Fig. 5d). Therefore, appropriate high-temperature treatment of plants effectively enhanced genome editing efficiency. Additionally, the endogenous promoters of *FmU6-6-4*, *FmU6-7-4*, and *FmECP3* of *F. mandshurica* were found to exhibit high activities in tobacco, birch, and poplar, suggesting their suitability for genome editing systems in other woody plants (Fig. 5e).

### Knockout of the *FmPDS1/2* reduced chlorophyll content

**Figure 6:**
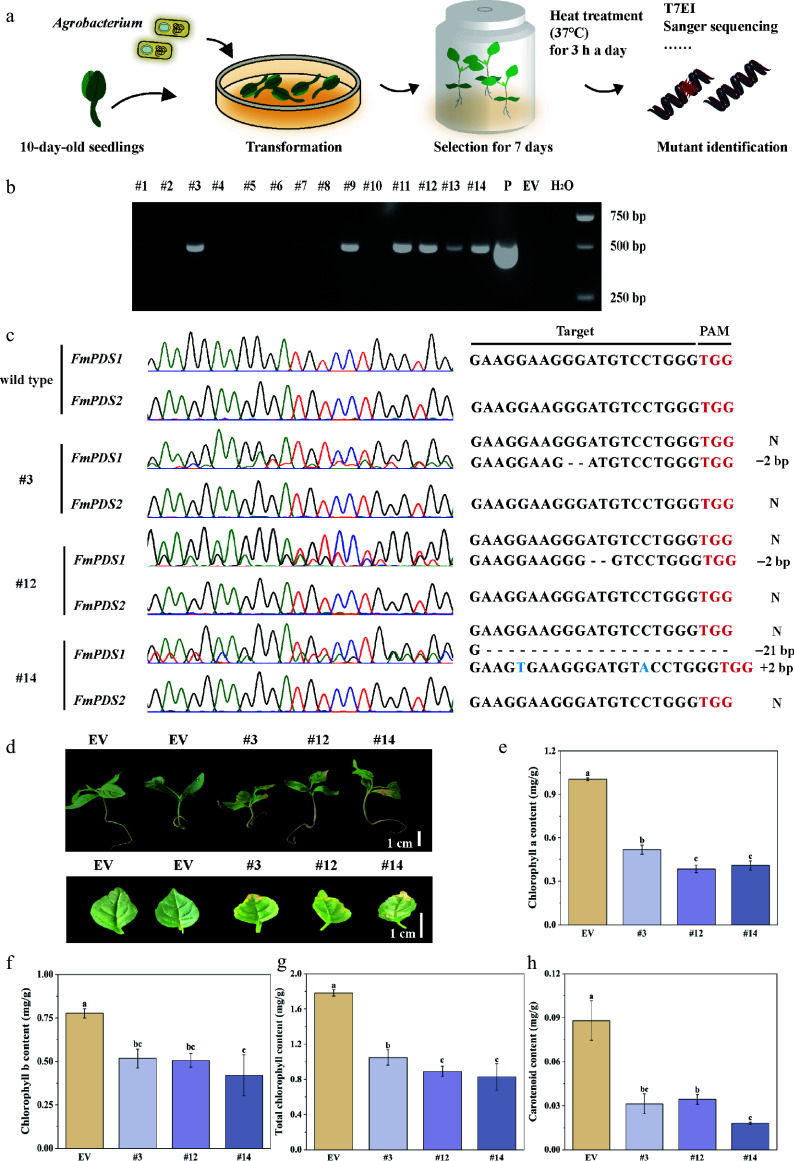
Transient transformation mediated CRISPR/Cas9 system in *F. mandshurica*. (a) Flow chart of the CRISPR/Cas9 system in *F. mandshurica* mediated by transient transformation. (b) PCR assay of the genomic DNA of six transgene-positive seedlings (#3, #9, #11, #12, #13, #14). P, plasmid; EV, empty vector. (c) Sanger sequencing results of *FmPDS1* and *FmPDS2* in mutations. N, without mutation. +, insertion, –, deletion. (d) Phenotypes of mutations, including the whole plant and true leaf. The scale bar represents 1 cm. (e) Statistics of chlorophyll a. (f) Chlorophyll b. (g) Total chlorophyll content. (h) Carotenoid content. Values represented mean ± SD (n = 3); different letters indicated significant differences (*p* < 0.05).

To quickly acquire gene-edited mutants for an initial analysis of gene functions, transient transformation of *F. mandshurica* seedlings was utilized to knock out *FmPDS* (Fig. 6a). After a rigorous antibiotic selection process, 14 seedlings were thriving. The expression cassette was successfully incorporated into the genome of six seedlings (#3, #9, #11, #12, #13, #14), according to the results of PCR validation (Fig. 6b). A 1,202 bp fragment of *FmPDS1* and 1,174 bp fragment of *FmPDS2*, covering the region targeted by gRNA4, were cloned and sequenced, respectively. For three of the six plants (#3, #12, and #14), overlapping peaks were observed in the target site region of *FmPDS1*, which is suggestive of chimeric and a 50% mutation efficiency (Fig. 6c). In contrast, the control (empty vector, EV) sequences showed no change. Further sequencing analysis revealed various mutation types and combinations. Two types of deletion patterns, such as a 2-nucleotide deletion in #3 and #12 and a 21-nucleotide deletion in #14, were observed. One type of insertion pattern was noted, with a 2-nucleotide insertion observed in #14 (Fig. 6c). However, no mutations were found in the target site region of *FmPDS2* (Supplementary Table S6). Functional redundancy and chimeric mutations are considered the primary reasons for the variegated phenotype observed in the mutant plants.

The three chimeric mutant plants exhibited light green overall compared to the control, with the appearance of small white or yellowish streaks and spots on their leaves, particularly at the leaf margins (Fig. 6d). Additionally, the mutant plants revealed a considerable drop in chlorophyll content in leaves (Fig. 6e–g). Interestingly, as compared to the control, the total chlorophyll content of #3, #12, and #14 decreased by 41.12%, 50.06%, and 53.56%, respectively. The carotenoid content in #3, #12, and #14 showed a substantial decrease, being only 35.59%, 39.10%, and 20.47% of that in the EV, respectively (Fig. 6h). These results offered information on the function of the *FmPDS* by indicating that its knockout in *F. mandshurica* seedlings caused visible phenotypic changes as well as alterations in chlorophyll content.

### Identification of *FmPDS1/2* edited transgenic *F. mandshurica* mutants

**Figure 7:**
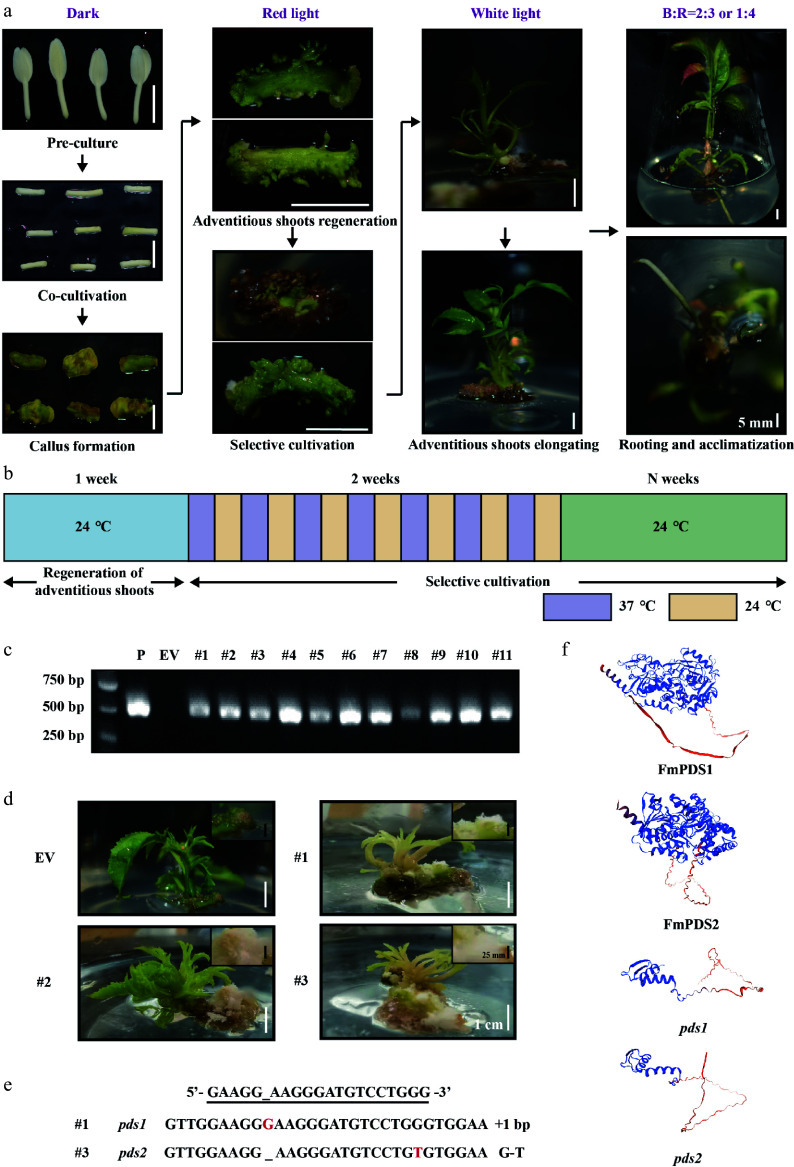
CRISPR/Cas9 genome editing of *FmPDS1/2* genes in *F. mandshurica*. (a) *Agrobacterium*-mediated genetic transformation process of *F. mandshurica* with hypocotyl explants. The scale bar represents 5 mm. (b) Schematic diagram of high temperature (37 °C) and room temperature (24 °C) alternating culture during selective cultivation. (c) PCR assay of the genomic DNA of seedlings. P, plasmid; EV, empty vector. (d) Bleaching phenotype of *FmPDS* gene-mutated plants of *F. mandshurica*. EV, non-edited plants. The white scale bar represents 1 cm. The callus was enlarged in the upper right corner. The black scale bar represents 25 mm. (e) Sanger sequencing results of mutations (#1 and #3). +, insertion; G-T, G substituted by T. (f) The 3D structures of FmPDS1, FmPDS2, *pds1* mutation in #1, and *pds2* mutation in #3.

The establishment and optimization of an efficient tissue culture regeneration system is crucial for generating mutants by CRISPR/Cas9. To explore the impact of light quality, various combinations were applied, including white and red light, as well as blue-to-red (B:R) ratios of 1:4 and 2:3, at different stages of the regeneration process using hypocotyls as explants. Findings revealed that the optimized light quality combination effectively increased the regeneration rate from 28.10% to 69.78%–70.19%, that red light was used for germination, white light was used for elongation, and B:R = 1:4 (G2) or B:R = 2:3 (G3) were used for rooting (Supplementary Fig. S5). To further ascertain the applicability of the optimized CRISPR/Cas9 vector in *F. mandshurica*, the *Agrobacterium*-mediated genetic transformation with hypocotyls as explants was used to produce stable mutants of *F. mandshurica*, combined with light quality optimization (Fig. 7a). Out of the 250 explants co-cultured for 3–5 d, 129 were encouraged to develop adventitious shoots within a week. During the first 14 d of selection culture, high temperature (37 °C) and room temperature (24 °C) were alternated to promote high gRNA and Cas9 expression and activity (Fig. 7b). After a month of selection and elongation culture, 11 of the 52 surviving adventitious buds elongated to form seedlings. These 11 seedlings (symptomatic/albino and non-symptomatic) were all confirmed as transgenic plants through specific validation primers YZ-sgRNA4-F/R (Fig. 7c). The control (EV) and other transgenic lines (e.g., #2) showed the whole green plants, whereas lines #1 and #3 exhibited albino and chimera phenotypes with albino stems and pale green leaves (Fig. 7d). Furthermore, #1 and #3 demonstrated slower regeneration and smaller, unexpanded leaves.

To confirm whether the observed albino phenotype was caused by *FmPDS1/2* gene mutations, sequencing of the target sequence regions of *FmPDS1* and *FmPDS2* was conducted. The sequencing results revealed homozygous mutations in the target genes (Fig. 7e). Line #1 had a one-nucleotide insertion in *FmPDS1*, while line #3 possessed a one-nucleotide substitution in *FmPDS2*, resulting in premature termination codons and truncated PDS proteins (Fig. 7f). Since the functional redundancy in the relationship between *FmPDS1* and *FmPDS2* in *F. mandshurica*, the mutants #1 and #3 exhibited chimeric phenotypes rather than complete albino. The flanking PCR fragments of five potential off-target sites were sequenced, which were obtained by using Cas-OFFinder (www.rgenome.net/cas-offinder) and searching the *F. mandshurica* genome database with the BLAST tool against the 20 bp target sequence of *FmPDS*. The results showed that none of the five examined off-target sites had any mutations (Supplementary Tables S5 & S7). Given the albino callus of the transgenic lines in Fig. 7d, it was speculated that simultaneous editing of two homologous *FmPDS* genes may have inhibited adventitious shoots regeneration in *F. mandshurica*, directly leading to failure in seedling formation. In conclusion, the CRISPR/Cas9 system is effectively applicable to *F. mandshurica*, with an editing effectiveness of 18.2% in this study.

## Discussion

**Figure 8:**
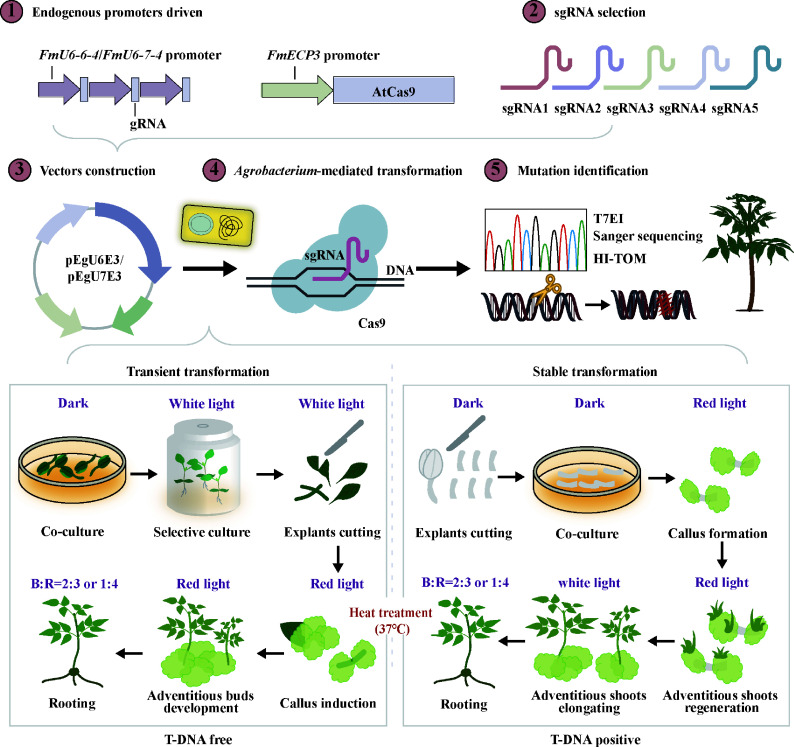
A generalized road map of CRISPR/Cas9-mediated genome editing in *F. mandshurica*. These practices all involve the identification and screening of endogenous promoters, sgRNA design, vector construction, *Agrobacterium*-mediated transformation, and mutation detection. Among them, transformation is categorized into transient and stable forms, depending on whether the T-DNA integrates into the mutant genome. Furthermore, various combinations of light quality and heat treatments are incorporated into the transformation process to increase transformation and editing efficiencies.

Despite the extensive application of CRISPR/Cas9 in various plant species, its utilization in woody plants is constrained by challenges in genetic transformation and heterozygosity, with well-established genome editing systems limited to tree species such as poplar and birch. The acquisition of genome-edited mutant plants in *F. mandshurica* has not been achieved to present, with the exception of the study by Lu et al., which reported a genome editing efficiency of only 8.6% in protoplasts^[45]^. Numerous studies have indicated that parameters including the promoters of sgRNA and Cas9, GC content and secondary structure of the target sgRNA, Cas9 codon, and temperature influence editing efficiency. In this study, a CRISPR/Cas9 system for *F. mandshurica* was developed and optimized through the identification of endogenous promoters, screening for highly efficient sgRNAs, and optimization of the optimal temperature. This system enabled the generation of genome-edited mutants targeting *FmPDS* (Fig. 8).

### The expression of gRNA induced by Pol III promoters

In gene editing, the expression of gRNA is typically induced by Pol III promoters, including *U3* and *U6*. Notably, *AtU6-1* and *AtU6-29* promoters in *Arabidopsis*, as well as *OsU6a*, *OsU6b*, and *OsU6c* promoters in rice, are commonly employed for genome editing in dicotyledonous and monocotyledonous plants, respectively^[46]^. The precise transcription initiation and stringent control over transcript length provided by *U6* promoters have facilitated their widespread adoption in the CRISPR/Cas9 system. However, recent studies indicate endogenous *U6* or *U3* promoters significantly enhance gRNA synthesis and editing efficiency. For example, the *VvU3*/*U6* promoters elevate the expression of sgRNA-targeted *PDS* by over 3-fold, compared to the *AtU6* promoter, resulting in a notable increase in editing efficiency in grapevine, which ranges from 14.65% to 22.10%^[28]^. The *ProLaU6-7* promoter enhances genome editing efficiency in larch from 4.92% (with *AtU6-26*) to 14.29%^[47]^. In contrast to the *AtU6-26* and *GmU6-5* promoters, the endogenous *U6-2*, *U6-6*, and *7S* promoters in white birch exhibit superior biallelic mutation rates^[25]^. Additionally, endogenous *LaU3*/*U6* promoters in white lupin have been reported to improve the efficiency of multi-target genome editing^[48]^. Notably, multiple *U3* and *U6* promoters often exist within the same species, exhibiting variations in activity and transcription efficiency. Among seven *CcU6* promoters identified in pigeonpea, the *CcU6_7.1* promoter exhibits the highest activity, while *CcU6_3*, *CcU6_9.1*, and *CcU6_9.2* promoters are ineffective^[29]^. In cotton, the *GhU6.3* promoter demonstrates superior activity compared to the *GhU6.1* and *GhU6.2* promoters^[27]^. There are significant differences in mutation efficiency among eleven *GmU6* promoters in soybeans, ranging from 2.8% to 20.6%^[26]^.

In the genome of *F. mandshurica*, seven *FmU6* snRNA genes were identified along with their corresponding promoters, successfully cloning four of these promoters (including *FmU6-3*, *FmU6-5*, *FmU6-6*, and *FmU6-7* promoters). It is generally accepted that each polymerase independently transcribes a unique set of genes, with Pol III specifically participating in the transcription of 5S rRNA, tRNA, and small RNAs. Nevertheless, some research has unveiled a synergistic interaction among the different types of polymerases in regulating gene expression^[49]^. Notably, Pol III, including *U6*, *7SK*, and *H1*, displays dual polymerase activity and has been shown to transcribe mRNA capable of translation, such as Luc^[50,51]^. The *U6* upstream promoter in yeast exhibits dual polymerase activity, being Pol II-specific *in* *vivo* but convertible to Pol III-specificity by TFIIIC^[52]^. Similarly, the mouse *U6* promoter generates transcripts sufficiently long to express multiple reporter genes, including *Luc*, *eGFP*, and *JRed*^[53]^. The integration of reporter genes (e.g., *GUS*, *GFP*, *Luc*) with transient transformation techniques facilitates a swift and effective evaluation of *U6* promoter activity. To date, a multitude of studies conducted on *Arabidopsis*, tobacco, roses, cotton, grapes, yams, and other organisms have proved the viability and efficacy of this methodology^[27,28,54−57]^. In this study, the reporter gene *GUS* was used to assess the endogenous *U6* promoter activity. These promoters exhibited significant differences in activity, with the *FmU6-7* promoter displaying the highest activity and the *FmU6-6* promoter showing the second-highest. Despite the high homology of the *FmU6* snRNA transcription sequences with other species, notable differences were observed in the promoter regions, apart from the conserved USE and TATA-like boxes (Fig. 1; Supplementary Fig. S1). It has been speculated that variations in the activity of different *U6* promoters within the same species may correlate with the number of CAAT-boxes and transcription factor (TF)-binding sites^[29]^. Additionally, large expression vectors result in genetic transformation in plants, which is complicated and difficult. Truncated promoter fragments of *FmU6-6* and *FmU6-7*, designated as *FmU6-6-4* and *FmU6-7-4* promoters, respectively, were identified to be 307 bp in length and contained USE and TATA-like boxes, which were sufficient to drive sgRNA expression (Fig. 2). Consequently, the *FmU6-6-4* and *FmU6-7-4* promoters were used to regulate the expression of target sequences within the *F. mandshurica* CRISPR/Cas9 system.

### The expression of Cas9 driven by Pol II promoters

Similarly, species-specific Pol II promoters demonstrate remarkable efficacy in enhancing Cas9 expression and increasing mutation frequencies. The rice *Ubi* promoter has been extensively employed in genome editing studies aimed at various traits in rice^[58]^. The grape *UBQ2* promoter has also shown effectiveness in driving Cas9 expression and facilitating gene editing processes^[28]^. To ensure efficient Cas9 protein expression in *F. mandshurica*, nine promoters derived from highly expressed genes with non-tissue and non-developmental stages specificity were screened, in addition to two promoters of the *EF-1α* gene. Notably, the *FmECP3* promoter exhibited the highest activity, being 5.48 times higher than 35S (Fig. 3). Furthermore, the identified *FmU6-6-4*, *FmU6-7-4*, and *FmECP3* promoters demonstrated activity in driving *GUS* expression in tobacco and other woody plants, including poplar and birch, indicating the potential applicability of the established CRISPR/Cas9 system in *F. mandshurica* to other woody species (Fig. 5e).

The integration of tissue-specific and inducible promoters into the CRISPR/Cas9 system has allowed for precise spatial and temporal regulation of genome editing. Efficient genome editing in maize has been achieved by employing the promoter of meiosis-specific *dmc1* to regulate the CRISPR/Cas9 system^[35]^. The employment of Egg cell-specific and early embryo-specific promoter (*DD45*/*EC1.2*)-controlled CRISPR as well as meiotic promoter (*MGE1p*)-driven CRISPR in *Arabidopsis* has facilitated the production of non-chimeric T1 mutants^[33,59]^. The *Yao* and *EF-1α* promoters, which are predominantly expressed in meristematic tissues and germ cells, provide advantages for mutant generation and have been applied to the CRISPR system across various species, such as citrus^[37]^, *Arabidopsis*^[34,60]^, and tomato^[36]^. Nevertheless, compared to *U3*/*U6* promoters, there remains a relative scarcity of studies focusing on promoters that govern Cas9 expression. Two *FmEF-1α* (*FmECP10* and *FmECP11*) promoters were tested to regulate Cas9 expression; however, the efficiency was not optimal. This could potentially be attributed to the fact that the explants were not derived from meristematic tissues. To elucidate the functions of genes with tissue-specific or developmental stage-specific expression patterns, tissue-specific and inducible promoters will be employed to regulate Cas9 expression in future investigations. Furthermore, studies have indicated that employing plant- or species-codon-optimized Cas9 enhances mutation frequencies^[30,61]^. In the present study, the Cas9 protein utilized was codon-optimized for *Arabidopsis*. To further improve mutation efficiency, it would be beneficial to apply *F. mandshurica* codon-optimized Cas9 in the CRISPR/Cas9 system.

### Impact of gRNA sequence on gene editing efficiency

The sequence and secondary structure of gRNA directly influence the efficiency of CRISPR/Cas9. Studies focused on genome editing in poplar indicated that gRNAs with a GC content ranging from 40% to 60%, along with purine residues in the last four nucleotides, are more suitable for target sites^[62]^. Target sequences with higher GC content exhibit relatively higher CRISPR/Cas9 knockout efficiencies^[46]^. The optimization of sgRNA by extending the duplex by approximately 5 bp and mutating consecutive thymine (T) nucleotides at the fourth position to cytosine (C) or guanine (G), significantly enhances genome editing efficiency^[63]^. To design sgRNAs with high efficacy and specificity (low off-target activity), various sgRNA models and design tools tailored for plant genomes, such as DeepSpCas9^[64]^, CRISPR-GE^[65]^, and CRISPR-P 2.0^[66]^, have been developed. In this study, the target sequences for CRISPR/Cas9 were designed by online programs (https://crispr.dbcls.jp/ and http://skl.scau.edu.cn/targetdesign/). The efficiency of the six selected sgRNAs was predicted using CRISPRedict and the two sgRNAs with the highest predicted effectiveness were selected to construct vectors. Both *in vitro* and *in vivo* experiments confirmed that sgRNA4 exhibited the highest activity, consistent with the predictions (Fig. 4).

The CRISPR/Cas9-based multiplex genome editing system targets multiple copies of the same gene, (homeo) alleles/paralogs, or different genes simultaneously, effectively addressing the challenges associated with obtaining mutants for target traits due to high genetic and gene redundancy. Currently, up to 107 genes are targeted simultaneously in plants^[67]^. A CRISPR/Cas9-induced genome editing system capable of targeting multiple sites and genes in both monocotyledonous and dicotyledonous plants has been established, which reveals that transgenic plants targeting eight sites simultaneously exhibited higher sgRNA levels than those targeting only one site^[46]^. In *F. mandshurica*, *FmPDS1* and *FmPDS2* shared 92.59% amino acid sequence similarity, suggesting similar functions. The two mutant lines developed in this work, lines #1 and #3, both exhibited chimeric albino phenotypes (Fig. 7). Line #1 had a 1-nucleotide insertion in *FmPDS1*, while line #3 had a 1-nucleotide substitution in *FmPDS2*, resulting in premature termination of *FmPDS1/2* translation. This means that *FmPDS1* and *FmPDS2* regulate the synthesis of chlorophyll and carotenoids in a dose-dependent manner, and albino in transgenic lines only occurs when both genes are altered simultaneously. Unfortunately, a double mutant of *FmPDS1* and *FmPDS2* was not obtained in this study. Notably, it was observed that callus tissues from transgenic lines were entirely albino, whereas callus tissues of the control were yellow, leading us to speculate that simultaneous mutations in *FmPDS1* and *FmPDS2* may inhibit adventitious shoot regeneration, preventing the obtention of albino seedlings. To verify this speculation and improve genome editing efficiency, a multiplex genome editing system for *F. mandshurica* would be developed by linking multiple sgRNA expression cassettes induced by the *FmU6* promoter to simultaneously target various sites of the same gene or multiple genes.

### Heat treatment significantly improves gene knockout efficiency

Temperature treatment influences genome editing efficacy. The Cas9 nuclease, produced from bacteria with optimal growth temperatures ranging from 35 to 45 °C, encounters partial inhibition of its activity under room temperature conditions (20–28 °C), which are suitable for most plant growth^[68]^. Empirical studies have shown that brief exposure of transformed plants to higher temperatures significantly increases nuclease activity. Specifically, in poplar and birch, heat treatment at 35 °C has been demonstrated to improve target site cleavage efficiency substantially^[42]^. Heat treatment at 37 °C significantly enhances the cleavage activity of SpCas9, sgRNA expression levels, and CRISPR-induced mutation efficiency in *Arabidopsis* and citrus^[69]^. Research conducted by Xiang et al. suggests that the increase in genome editing efficiency induced by high temperature is attributed, at least in part, to the upregulation of sgRNA expression and the enhancement of Cas9 protein activity^[70]^. To ascertain the optimal temperature for the CRISPR/Cas9 system in *F. mandshurica* and investigate its fundamental mechanisms, the activities of the *FmU6-6-4*, *FmU6-7-4*, and *FmECP3* promoters were quantified, as well as the cleavage efficiency *in* *vivo* across a range of temperatures (22, 28, 32, and 37 °C) (Fig. 5; Supplementary Fig. S4). Consistent with previous findings, the results revealed that high temperature, 37 °C, effectively upregulates the expression of sgRNA and Cas9 in *F. mandshurica*, thereby promoting the cleavage of genomic DNA.

### Transient transformation and stable transformation mediated CRISPR/Cas9 genome editing

Genetic transformation has provided a powerful tool for studying gene functions in woody plants and for the directional improvement of desirable traits^[71,72]^. Plant transformation techniques primarily include direct (such as physical and chemical) and indirect (like biological) approaches. Currently, *Agrobacterium*-mediated delivery of T-DNA is the preferred method for plant transformation. Transformations are categorized into transient and stable types, depending on whether the introduced foreign gene can be a stable inheritance. Progress in applying CRISPR/Cas9 technology to woody plants has been relatively slow, with the inefficiency and complexity of stable transformation systems serving as a key impediment to its utilization in trees. Notably, the transient expression of CRISPR/Cas9 in cacao has identified *TcNPR3* as a defense regulator^[73]^. A novel method, named CPDAT, enables the generation of CRISPR-edited plants without the integration of foreign DNA, in which *Agrobacterium*-mediated transformation is used to introduce T-DNA encoding gRNA and Cas9 into birch cells via transient transformation, achieving genome editing in 80% of the cell lines^[74]^.

However, the genetic transformation system for *F. mandshurica* still requires refinement. The transformation efficiency is low and the process is lengthy when hypocotyls and embryogenic callus are used as explants^[43,75]^. In this study, *F. mandshurica* mutants targeting *FmPDS* were obtained by combining transient transformation with seedlings and stable transformation with hypocotyls as explants. Through transient transformation, chimeric mutants targeting *FmPDS1/2* were rapidly created, and preliminary evidence that the deletion of these genes inhibits chlorophyll synthesis was provided, consistent with previous findings (Fig. 6). By adopting transient transformation, the transformation time, including mutant identification and functional studies, was reduced to three weeks, significantly enhancing efficiency. Furthermore, mutants were successfully generated via the stable genetic transformation, validating the high effectiveness of our established genome editing system mediated by CRISPR/Cas9 in *F. mandshurica*, achieving an editing efficiency of 18.2% (Fig. 7).

### Generation of DNA-free woody plants mediated by CRISPR

Given current genetically modified organism (GMO) regulations, transgene-free (non-GMO) genome editing has emerged as a preferred approach. CRISPR/Cas9-mediated crop mutants produce non-transgenic progenies through sexual reproduction and screening of segregating populations; however, this method does not apply to perennial woody plants. As an alternative, CRISPR/Cas9 ribonucleoprotein (RNP) is delivered into protoplasts via transient transformation or particle bombardment using plasmids that encode sgRNA and Cas9. Nonetheless, pre-assembled RNPs and a comprehensive system for protoplast preparation and regeneration are necessary, both complex and time-consuming.

Transient transformation-based CRISPR/Cas9 provides a quick and effective DNA-free process for obtaining mutants. Tobacco leaves are used as explants to generate non-transgenic mutant plants without sexual segregation through an *Agrobacterium*-mediated transient genome editing system, achieving an editing efficiency of 47.5%, with 17.2% confirmed as non-transgenic^[76]^. Similarly, in the CPDAT method, white birch explants derived from transient transformation are employed to induce callus and adventitious buds, and edited plants are screened through sequencing, resulting in an editing efficiency of 80.00%, with 7.69% of the plants being DNA-free^[74]^. Recently, an efficient transgene-free CRISPR/Cas9 method using transient transformation has been devised for poplar^[77]^. Using an *Agrobacterium*-mediated approach, callus tissues and plants are transformed with a base-editing construct targeting *ALS* for positive selection and *CCoAOMT1*. Explants were stimulated to generate shoots on the medium containing chlorsulfuron, with 49% of the newly formed buds originating from transient transformations devoid of T-DNA and 7% identified as non-chimeric mutants. In the present study, whole seedlings were employed as explants, and multiple mutants were obtained via temporary expression of sgRNA and Cas9. Sequencing results indicated that plants generated via this strategy were inherently chimeric (Fig. 6c). Isolating and regenerating the chimeric mutants may provide an effective means to separate chimeras and obtain homozygous transgene-free mutants (Fig. 8). However, due to the lack of a supporting tissue culture system, the induction of transgenic DNA-free plants was not pursued.

## Conclusions

This study demonstrates the critical significance of species-specific promoters, sgRNA design, and thermal regulation in optimizing CRISPR/Cas9-mediated genome editing efficiency in *F. mandshurica*. A set of endogenous *FmU6-6-4*/*FmU6-7-4* and *FmECP3* promoters was discovered in *F. mandshurica,* which effectively drive sgRNA and Cas9 expression, respectively. *F. mandshurica* mutants targeting *FmPDS* were successfully acquired by combining transient transformation with seedlings and stable transformation with hypocotyls (Fig. 8). The CRISPR/Cas9 system, particular to *F. mandshurica,* provides a flexible platform for functional genomics research and enhancement of traits in this species, facilitating the incorporation of remarkable characteristics. Notably, the endogenous promoters identified in this study displayed conserved activity in poplar and birch, demonstrating broad application of this editing platform to other woody species. This work establishes a transformative framework for implementing CRISPR/Cas9 technologies in forest tree molecular breeding programs.

## SUPPLEMENTARY DATA

Supplementary data to this article can be found online.

## Data Availability

All data generated or analyzed during this study are included in this published article and its supplementary information files.
