# Supplementary material for: Development of an endogenous promoter-driven CRISPR/Cas9 system for genome editing in Fraxinus mandshurica
Source: For Res (Fayettev). 2025 Aug 4;5:e016. doi: 10.48130/forres-0025-0016 (PMC12441911; doi:10.48130/forres-0025-0016)
Supplement: Supplementary file 1 — Supplementary data to this article can be found online. [file FR-2025-5-0016-Supplementary.zip › 10.48130_forres-0025-0016-Suppl-FigureS2.pdf]

Fig. S2 Genomic sequences of *FmPDS1* and *FmPDS2*. Color-coded sequence information for *FmPDS1* and *FmPDS2* sequences (red = sgRNA4 sequence, blue = sgRNA6 sequence, green = PAM sequence, purple = Primer sequences for PCR amplification, Orange = Primer sequences for HI-TOM, and lowercase letters represent PCR amplification sequences).

>FmPDS1

CAGAGAGTCTCAATTTTTGGAAGCTAATAGAGTGGACGAATGGATAGCATATTCTTACCTACAGCA
AACAATTGCGTAACTACGAGTGCGGCCAAACCAACGAACCATTTACAACTCCCACCCAACCCCAC
CCCACCCAACCCTGTCATCTTGGCTCTTAAGATGCCACACCACCATAACTTAGTGCTTGTAAACTCT
ACCAATTATCTTTCTTGAAAATACCCTGTTCAATTTTCACCTTTTCAGTTGCTGAATTAAGAGCTCG
GTTGAGTGTCGCCTCCTTTTGATTTACCCGGAAGGTAATTTTACAGAATTCGCACCGCCTTTTTTAA
AAAATATTTGGCGATGAGCTGGTTTTGTTAGAAAAAGTAAAATCTTGAGTTGGGATTGTATAGAGA
AAATATTAATTTGAGGTTAATTGATAGTGTGTTCTGAAGGTTGTTGAAAAGCTAATGCCTTTTAATT
TTCTGTCTATGGGTTCTTTGATTCGAGTTACGCTTCGCAATTCTGGAGTTCATGTATAAACTAAATA
AAATATTTGGACTTTTTGAGGTAGAATTCTGAAAGGTTTAAAATTTTAGGATTCCATGTTGTTGTCT
AAATTTAAGTATTTATGCAAATTATTTGCTTTTAGGATTCTTGATATGGATACATTTTTAAACTATG
ACACAAATTTATAGTTGCAGGGCTGCAATTTTTGAGCTTGTTTAATTCATAAACATTGTTGTAAGG
AAATCTTGAATTTTCGAAATGTCCCAATTTGGACATGTTTCTGCTGTTAATTTGAGAGGGCAAAGT
AATCCTTGGAGCCCTCAGTCTTGGAGATGCTGCTGTCTTGAAGGGAAAATGAATACCCTATCATTT
GCAAGTAGTGATGCTATGGGTCATAAATTGAAGAGGCCGTCTGCACATGCTTTGATGATGGATTCA
AGAAGAAAAGATATGTCCCCTTTGAAGGTCTGGCTCCAAAGAATTCTTAATTGATTAGGGAATTTC
TGAAATTTTAGTGAACGAAGAAACACATTGATTTTATGTTTATTATATAGGTTGTTTGTATTGACTA
TCCAAGACCGGCAATTGAGAATACAGTCAATTATTTGGAAGCTGCTTATCTATCCTCATTGTTTCGT
AATTCTCCACGTCCAACCAAACCATTAAAGATAGTCATTGCTGGTGCAGGTGGGATTAACATGTTC
TCTTTATCTTTTTGTCATTTATTCCTTTTGTGTTTCTTTAGGACAGAGATTATAATTCCAGCGAATTT
GCACATTCTTATACACTGATAAAATTTagttagctgttagtgctttgcgcaatttggtgttgagcttagttagaaatttagaatgtaatttataatt
attcatctggaggacccacacttcccaattgaattctacagttcatgttttgctctttattttttccttacaagttatttttggatatatagtgagaaactattttatatactacac
tgaatccagtcaaaagttttatctaatgaaatcattcaccgagtgcgatgaattgaactactggatgcttgcaggtttggctggtttgtctactgcaaagtacttggcaga
tgcaggtcataaaccgatattgttggaaggaagggatgtcctgggtggaaaggtttataaatgcttccttgcttatgttcttctcaaaactatatttttatgtttaatatgtttc
ttctgtgaactcgcccctcgttgtccaggtggctgcatggaaagatgatgatggagactggtatgagactggattacacatattttgtaagtttgagaactcagttgcaa
ctttaaataccttatattatgcatcaaatgacttgtcaatttgattatttattttctttaagttggggcgtacccaaatgtgcagaacctatttggagagctaggcattaatgat

cggctacagtggaaggaacattctatgatatttgcaatgccaaacaagccaggggagtttagccgatttgattttcctgaagtcttacctgcaccattaaatggtgagtt

gtttattgttatcattggagaagctaagtaactgaaatgagaaaaacctccagttactttcatacaaccatgcctttaatatttgagacgaaacacactatgggttttcactt

attccttgcataagggtctgtaggtggtcatgcctttaatgtttgagacgaaacacactatgggttttcactcattccttgcataagggtctgtaggtgatgcgcaaggg

ccagtagctatccattcatgagatgtacaacacatttttctactcgattttttcctttgttttaaattttatattggcaggaatatgggcaatcctgaagaacactgaaatgctt

acttggccagagaaagtcaagtttgctattggactcttgccagcaataattggtggacaatcttatgtggaggctcaagatggtataactgttaaagactggatgagaa

agcaagTATGTAGAGTTGCATTGGTTGACGAGACCTGAATATTGCTAGATTTTCTCAATTTTTCTTTAC

AATAGAGTTGCACGAGTATCACTTGGCCAGTTTTGTCCTGACGAATTAATACATTTAATTCGTTTCA

GGGTATTCCAGATCGAGTTACTGATGAGGTGTTTATTGCCATGTCTAAGGCACTGAACTTCATTAA

CCCGGATGAACTTTCAATGCAGTGCATTTTAATTGCTTTGAATCGATTTCTTCAGGTAAATGGGCTC

ATCTCTCCTACGGTGTTGTTGATATCCTTCAATAAATATGTACATCGTGTGATTGAAGAAGCAAGA

CTCAATTATGAATTTTGCACAGTACGTCGCTGGGTGCTATATGTACACTTCTCATATTGACTATTAT

TCGGCTCGATCATTCAGTCTTCTTTTTACAACACAAAACAACTGAGTTTAATGATTGTAGCATCTCT

AACATGCTATATCTAATCAATAAAAAAATTACTTTGCTGTATTATTTACTTTGGTTCTTCAAATGAT

CAATTTACATATCTTTGAATTTGTATAAGAGTAACACTCATGATGAGCTCTCCATAATATTTTAATA

AAAATCTACAGGAGAAGCATGGTTCAAAGATGGCTTTTTTGGATGGCAACCCACCAGAGAGACTT

TGCATGCCAATTGTTGACCATATTACTTCACAAGGTGGCGAAGTCCAGCTTAATTCACGAATACAA

AAGATTGAGCTAAATAAAGACGGTAGCGTTAAGAATTTCATGCTAAATAATGGGAGTACGATTGA

AGGAGATGCTTATGTATTTGCAACCCCTGGTAGATTATTAATTTCTATAACAACTCATATATTGATT

TTTTAGTCTAAACGTTTCCGCGATTGCTAATTTGCTGTAATCCTATCAGTTGATATCCTCAAGCTCC

TTCTGCCTGAGGACTGGAAAGAGATTCAATATTTCCAAAAATTGGAGAAATTAGTTGGAGTTCCAG

TTATAAATGTTCACATATGGTAAGTTTTGGATCTTTTTTTCATTTTATTTGACGGTTATTTTCTCTGC

CTACGATGATCTGACTGAGTAATGTAGAATATACTCATAAAACTATAGTCATTATGTCATGCAAGC

TAGGACTAATTTGCCTTCTTCGTTGATATTTGGACTTTTTTTATTCATTCGATTTTAAGGTTTGCATC

TTAATTGCAGGTTTGACAGAAAATTGAAAAACACGTATGATCATCTACTCTTCAGCAGGTCGTTGT

CATAAACATTCAGTTCTACTAAATTATGTTACTATTTCAATTGCATACTTAATGTAGCATAGCGGAC

ACACACGGCTTTCATATTCCACTGATTATAAACCTACTAATTCTTCTTCTTCTTCTTCTTTTTTTT

TTGAAGTTTTTGCACAAGTGTTGTCTGTGAGTTGCCATAGCATTCTGTGTTCAATCTTATGAATTTA

TTTCATAATTTCTTTTAGGATAGCCGCCTTCAATTCTGTAGTTACCTACAATAATAAGTTTACGATA

CCTTCAATCTTTTTTACTTGTCCAATTTCATTTTCTGTGAGCATATCGCCCCTATTTGACAACCATTA

TTTTAAAACTCTTTAATTCTCATATTCATTTTCAACAACATATTTATACAATAAAATATTTTGCACG

TAAACCCAAAACATTTCTCTACTTTTTAACTATACATTATCCAAAAAATCATTATTCAAAACTCCAT
TTTTTTCAAAATAATGGCCATCAAACATGGGCTTAATGAGAAGAACTAATACACTTATGGCATGTT
TAGTAGGGACGTAGAATAACAGTAGACATTATAAACGAAAAGAAGAAGCTAAAATTACAATTACA
TGTATAAATTATAATTAAATGTCCCACAAATTCTTATTTATTTGCTGATGTTTATGCTCTCCGTGGT
ATTTAAGGCTAAAAGAGAAAATTTCATCAAATCCTGATTGACTTATTTTCATCTAGTTGTGTAATAC
CTTTTCTGCCTGATTCTGAGATTAACATTTTTCCTTTTCTTTTTTTGCCATTTAATGCAGGAGTTCAC
TTCTCAGTGTATATGCTGACATGTCTGTAACTTGTAAGGTACTAACGGCATATTATCGTATTTTTTA
CTATTTTTATACTGGCTTGATCTTTATAATTAGTTTTGTAATTGCCATAAAATTCATTTAAGAATAT
ATATATTTTCTAGTGCCGTGGTTGTGTAATGCAAATGATGTAGATTAAGGTTTTTTCTCATAAATAT
GAATGATATAATAGTAAACTTTCCCAGTTGATATTTTTACGAGGGCTTACTTCGCACAATTTGAGT
ATTTTCTATTTATATTTGATCTCGTGAGGGGCAATTATACGTCGTCTTTGTTCCTACTCGGATTTTGT
TAACTATACTTTTATGGTTGCCCAAATGTTCTTACAGGAGTACTACAATCCTAATAAATCTATGCTG
GAATTGGTTTTTGCACCTGCAGAAGAATGGATCTCTCGTAGTGACACAGAAATAATCGATGCCACA
ATGAAAGAACTTGCAAAACTCTTTCCTGATGAAATTTCTGCTGATCAGACCAAAGCAAAAATAGTG
AAGTACCATGTTGTTAAAACTCCAAGGTTAGACATGAATCGATACTTACAGTAGCAATCTCATGCT
TTAGCCCTTTACTGGTAGATGATGAGATCTTTATCTTTCATGTAAATATGGCCATGCACTAGCTGAT
GATGACAGCATTCAACTCTCTACATGAAACTTTTTCACTGTTTTGTTGTTTCATAAGTCAGATTTTTT
GCTGTACTGGACATCGGTTACAACATTAAATTTTGGTCAGGAGGAACAGATACAAGTGGACATAA
CTGGACAGCATTAAGGATGAAAAAGAAACTAAAATCTTGTTCATAATCTAGTCAAATCTTGTCTTC
TAATTAGCATACCGGACATGCACTATGGTATATTTTGTACGGAGGATTGCATAACATTGGACAATA
CTATCATGTTCAGTACTCCCCGTTTATTGAATTCAGACTGAGCTTCTCTCCTCTAGGTCTGTCTATA
AAACCGTACCTGGGTCTGAACCTTGCCGCCCCTTACAAAGATCTCCTATAGAAGGATTCTATTTAG
CCGGTGATTACACAAAGCAAAAGTACTTGGCTTCGATGGAAGGTGCTGTTCTATCGGGAAAGCTTT
GTGCACAAGCCATTGTGCAGGTGATGTTTCACTCACCTCGTTTCTCTCTCAGACGCATACATTAGTC
AAACGAATCTTATATCCAAACTTCAATCTCTTTTAAACACTTTGCAGGATTATGAGTTATTGGCTGC
AATAGAACACAAGAAGTTGGCAGAGGCAAGCCTTGTTTAACTTTTCTAAATTGAAATTCGGGATTT
TGCTATTCAATTGTATAGAAAAATATTGTAAAGATGGACGAAAGCCATGCAGACCGTATACTATTT
ACATATTGTGTGAATCAAAGAAATAGAAGATGATAATCTACTGAAGTAGCAAAACTACCACACAT
TGGTTTGCCGTTCTAGTACAAGTTCATTTTCAGT

>FmPDS2

GTGTAACCACTCGCCCCACCCCGTCATCTTGGCTTTCATGCCACACCACCATAACTATGCTGGTAA
ACTCCACCCATTATCTTAGTTCTTGAAAATACCCTTTCAATTTTCACTTCTTAAGTAGCTGAATTAA
GAGTTTGATTTGCCCGGAGGTAAAATTCTCTAATTTAACAGAACTTGAATAGCTTTTTTTCCTCGTT
TTCTTTAAATCATTTGGTTATGAGCTGGTTTTCCTAAAAAGAAACAATCTTGAGTTGGGATTTTATT
GGGAAAAGATTAGTTTGAAGTTAACTGATAGTGTGCTCGGAAGGGTGTTCAAAAGTTACTGTTTTT
TATTTTTCTGTGTTTGGGTCCTTCATTCGAGTTACATCGATTTCTGTGCAATTCTGGAGTTCTATAAA
CAAACTAATATATCTGAGTTTTTGAGGTAGAATTTTGAAAGTCTTAATAGGTAGGAATTTCTTGTTC
TTGTCTATATGTAAGTATTTATGCAAACTATTTGCTTTGGGGGTTCTTGACATGGATAAAAAAGATT
AATTTGAAGTTAACTGAGAGTGGGTTCTTCGTTTCGAGTTACATCTATTTCTTTGCAATTCAGGAGT
TTTATAAACAAATTAATATATCAGGGGTTTTGAGGTAGAATTTTGAAAGGCTTAAAAGGTTAGGAT
TTCATGTTTTCTAAATTTAAGTATTTATGCAAACTATTTGTCTTTGGATCCTTGATATGGATACATTT
TTAAGTATGACGTATATTTAGAGTTACAGGGCTGCAATTTTGGAGCTTGTTGAATTCATAAACATTT
TTTTTGAAGAAATCTCGAGTTTCGAAATGTCCCAATTTGGACATGTTTCTGCCATCAATTTGAGAGG
GCAAAATAACTTAAATAATCTTTGGAGCTCTCAGTCTTGGAGATGCTACTGTCCTGATGGGAAAAT
GAATACACTATCATTTGCAAGTAGTGATGCTATGGGTAGTAAATTGAAGAGTCCTGCTGCTCATGC
TTTTATGACGAGATCAGGAAAAGATGTGtccccttttgaaggtatggccccaaagaaatgttaaatgattaggggtttttgaaatgttaatga
aagaagaaacacattgatgttatgtttgttatataggttgtttgcattgactatccaagaccagggattgagagttcagtcaattatttggaagctgcttatttatcttcaaca
tttcgtaactctccacgtccatacaaaccattaaagatagtcattgctggtgcaggtgggaataacatgttttctttatctttaaaattatttttcccttttctgtttcttgagcaa
atagattaaaattctcgtgaattcacatacccttttacaccacgactaaaatgtagttagctgttagtgcttattcggtgaagaactttgtttagaaatttaagatgttatttttt
agttatttatctcaagtatccccaattcccttgggaataagtacagttgtgtcaaaagtaaaagtgatatcaagtatattcctcaacataggatttctttactagccttttcga
caaatccaatcaacgggttgctttttaatggatgtttttgtcaataggattctatagttgatatttgcatttatatatatttttctttctcacgttgtactttttggaactatagttag
aaactaatataaatactacactgaatttagtcaaagatttatttaatgaaatcatttacctagtgctgatgaattgaactactggatgcttccaggtttggctggtttgtctac
tgcaaagtatttggcagatgcaggtcataaaccgatattgttggaaggaagggatgtcctgggtggaaaggtttataaaatgctcccttgcttacgttattctcaaaact
attcttttatgcttaaagtgtttcttcactgaatttgtccctcgttgtgcaggtggctgcatggaaagatgatgatggagactggtatgagactgggttacacatattttgta
agtttgagaactcagttgcaactttaaatactttgtattatgcatcaaatcacttatcaatttgattatttattttctttaagttggggcttacccaaatgtgcagaacctatttg
gagagctaggcatcaatgatcggctgcagtggaaggagcattctatgatatttgcaatgccaaacaagccaggggaaTTTAGCCGATTCGATTTT
CCTGAAGTCTTACCTGCACCATTAAATGGTGAATTTTTTTTTATTATTTAATTTAATTTTTATTTTTT
TTAATCCTTGGAGAAGCCAAGGATTTGAAATGAGAAAAAACCTCTAGTTAGTTTTCATACAACTGT
GCCTTTGATATTTGAGGCAACGCTCAAGATAGATTTGCATTTATTTATTTCATAAGGGTCTGTAGGT

GCATAAGGACGTGCACATTTTTCTACACTATTGTTTTCTTGTGTAAAATTTTATTGTTATTGGCAGG
AATATGGGCAATCTTAAAGAACAGTGAAATGCTTACTTGGCCAGAGAAAGTCAAGTTCGCTGTAG
GACTCTTGCCAGCAATATTTGGCGGACAGTCTTATGTTGAGGCTCAAGATGGTATAACCGTTAAAG
ACTGGATGAGAAAGCAAGTACGTAGAGTTGCATTAATGAACGAGACCTGAATATTGCTAGATTTA
TCTCAAATTTTCTTTACAACAAAGTTGCATTAGTATCTCTTTGGCATTTTTTTTTGGGCGTGTATCT
ATAGCTATTATTTTCCAGTTTTGTCCTGAGAAATTAAGGCATTCAATTCGTTTCAGGGTATTCCAGA
TCGAGTAACTGATGAGGTTTTCATAGCCATGTCTAAGGCACTGAACTTCATTAACCCTAATGAACT
TTCAATGCAGTGCATTTTAATTGCTTTGAATCGATTTCTTCAGGTAGACTAGCTCTTCTCTCATTCA
GAGTTGTTGATATGCTTGAGTAATTATGTATATCGTTTGATTGAAGAAGCAAGACCAAATCGAACT
TCACCTGGAAAGCCGCTAGGTGCTATATTTACAGTTCTCACATTAACTGTTATTTGACTCGATGAAT
CAGTCTTCTTTTTAGAACACAAAACAACTACATCTATGGTTGTAGCATCTCTAACATGCTCTATCTA
ATCAACAGGATAATTACTGTTTTGTATTTACTTTGGTTCTTCAAATTATTAGTTTACATATCTTTATA
TTCGTATAATAGTACTGTTCATGATATGAACATTTAATAAAAATCTACAGGAGAAGCACGGTTCAA
AGATGGCTTTTTAGATGGCAATCCACCAGAAAGACTTTGCATGCCAATTGTTGACCATATTACTT
CACAAGGTGGTGAAGTCCGGCTTAATTCACGAATACAAAAGATTGAGCTAAATAAAGATGGCAGT
GTTAAGAACTTTGTGCTAAATAATGGGAGTACGGTAGAAGGAGATGTTTATGTATTTGCAACCCCC
GGTATATTATTAATTTCTATAACTAATCATATATCGATTTTTTAGTCTAAACCTTTCCATGGTTGCTA
ATTTGCTGTAATCCAATCAGTTGATATCCTCAAGCTCCTTCTGCCTGAGGACTGGAAGGAGATTCA
GTATTTCCGAAAATTGGATAAATTAGTTGGAGTTCCAGTTATAAATGTTCACATATGGTAAGTTTT
GGATCTTTTTTTCAATTTATTTCTTTGCATAGGATGATATGAGCGAGTAATGTAGAATACTCACAAA
AATATAGCCATTATCTCATGCAAGCTAGGACTAATTTGCCTCCATCATTGATATTTGGATTTTCTTT
TATTCGAATGATGATTTTGCATTTTGATTGTAGGTTTGACAGAAAACTGAAAAACACATACGATCA
TCTACTCTTCAGCAGGTTGTTTTCATAAACATTCAGTCCTATTGAATTATATTATTTCCTCAATTTCA
TACTTAGATTTACCATAGAGGATCAATAATGCTCTACATATTCCATTGATATATAAGCCTACGATTC
TTTTCTTTTAAATAATTATTGCACAAGCATTCTGTGTGAGTTCCCATGTCTCCACCCCTGTTCTGTAG
TTACCTAAAATATTAATTATCAATCCCTTAAATTTTTTTGGGCTTTCCCAATTCATTTTCTAATATAC
TTATAGCATGTTTGGCAGGGACAAATGTATAGCGTCAGACATTATAAGGAAAAAAAAAAAGAAGA
TAAAATCATTTTACAGTTACATGTATAAATGATAATTATTAAAACAACCCACAAATTCTTATTTATT
TACTGATATTTTATGCTTTCTGTGGTATTAGAGGCTACAAGAACACATTTTGTCAAATCCACTTTTA
GTTTTTTCATCTAGTTGTGTAATACCATTTTTGCCATTCTCCATTATTCACTTTGTTTTTTCCATTCAA

TGCAGGAGTTCACTTCTCAGTGTGTATGCTGACATGTCTGTAACTTGTAAGGTACTAACTAGATATT
ATCGAATTTTTACCAAAATTTTATACTGGCTCCAACATTTTTATTATTTCTGTTAACAGCAGGGAAG
ATAAGTTTGTATTAATTTTGTTACAGCCAAAAATTGATTTTTAAGATTTTCTTTTCCTCAAACGATG
GTTGTGTTGGGTGCCACGACCGTGTACAGGAAGCTGGTGACTGTAATATCCTGATGCTGTTGCTAT
AACAAGATTAATCTCTTTGGATATCATGTAAACGATGAAGAATTAAGGTTTTTAAATATGAACAAA
ATAATAGTAAATTTCCAGTTGCTTTTTCAAGGGATTCACCTAATTTGACTGTTTTCTATTTCATATTT
GATCTCGAGAAAGGGATTGATGTATCATTTTTATTCCTACTCGGCTCTTGTTAACTGTACTTGTATG
TTTCTCGTAATTTTTTTACAGGAGTACTATGATCCTAATAAATCTATGCTGGAATTGGTTTTTGCGC
CTGCAGAAGAATGGATCTCTCGTAGTGACACAGAAATCATTGATGCCACAATGGAAGAACTCGCA
AAACTATTTCCTCATGAAATTTCTGCAGATCAGACCAAAGCAAAAATACTGAAGTACCATGTTGTT
AAAACTCCAAGGTTAGCCATTAACCGAAATTTACAGTAGCAATTTGGTTAAGCCTTTCATTATTTT
GTTGTTTCATAACAAAGATTCTTTGCTGCACTGGACATACAGTTACAGCATTAAATTTTGTTCCCGT
TGTCCTTAAAACCTGTGGAAACTTTACAACATGTTTGGTAAGGAGGACCGCACAAAAGAGGACAT
AACTGGACAGTAATAAGGAGGAAAAAGAAGCTAAATTCTTGTTCGTTATGTGTCGAATCATGATC
CAGTAATGCACACCAAACATACACTATCATATATTTTGTAGGGAGGATTGCATAATATTGGACAAT
ATTGATGATGAAAAAGAAACTAAAATCTTATTCGTTATACAATCATATTTAGTATTGCTTGATTATT
GACTTCAGCATAACTGAGTTTCTCTCCTCTAGGTCCGTCTATAAAACCGTTCCTGGCTCTGAACCTT
GTCGTCCCTTGCAAAGATCTCCTATAGAAGGATTCTATTTAGCTGGTGATTACACAAAGCAAAAGT
ACTTGGCTTCGATGGAAGGTGCTGTTCTATCTGGAAAGCTTTGTGCACAAGCCATTGTACAGGTGA
TTTTTCACTCGCCTCGTTTCTCTCTCAGATGCATACACTAGTCACAAGAACGAACCTTATATCCACA
AATCATTTTGCAGGATTATGAGTTGTTGGCTTCAAGGGAACCAAAGAAGTTGACCGAGACAAGCC
TTGTTTAACCTTTATAAATTTGAAATTCGGGATTTCTTTTTCCAATTATATTTATAGAAAAATCATG
AGAAGATGGATGAAAGCCATGTAGACTATACAATTGACATACCGGGTGAATCAAAGAAATGTAAT
CAAAATGATGATAATCTGTTCACGTGGCTGATTATACAATTTACGCTTCCCCATATTTTTC
