# Supplementary material for: Development of an endogenous promoter-driven CRISPR/Cas9 system for genome editing in Fraxinus mandshurica
Source: For Res (Fayettev). 2025 Aug 4;5:e016. doi: 10.48130/forres-0025-0016 (PMC12441911; doi:10.48130/forres-0025-0016)

Fig. S3 Multiple alignments of PDS protein from different species. Fm: *F. mandshurica*, Oe: *Olea europaea*, Dh: *Dorcoceras hygrometricum*, Sl: *Solanum lycopersicum*, Nb: *Nicotiana benthamiana*, Pt: *Populus trichocarpa*, Vv: *Vitis vinifera*, At: *Arabidopsis thaliana*, Os: *Oryza sativa*, Zm: *Zea mays*, and Ta: *Triticum aestivum*. The phylogenetic tree is drawn with 1000 bootstrap trials using the Jones-Taylor-Thornton (JTT) model and the neighbor-joining (NJ) method in MEGA-X.

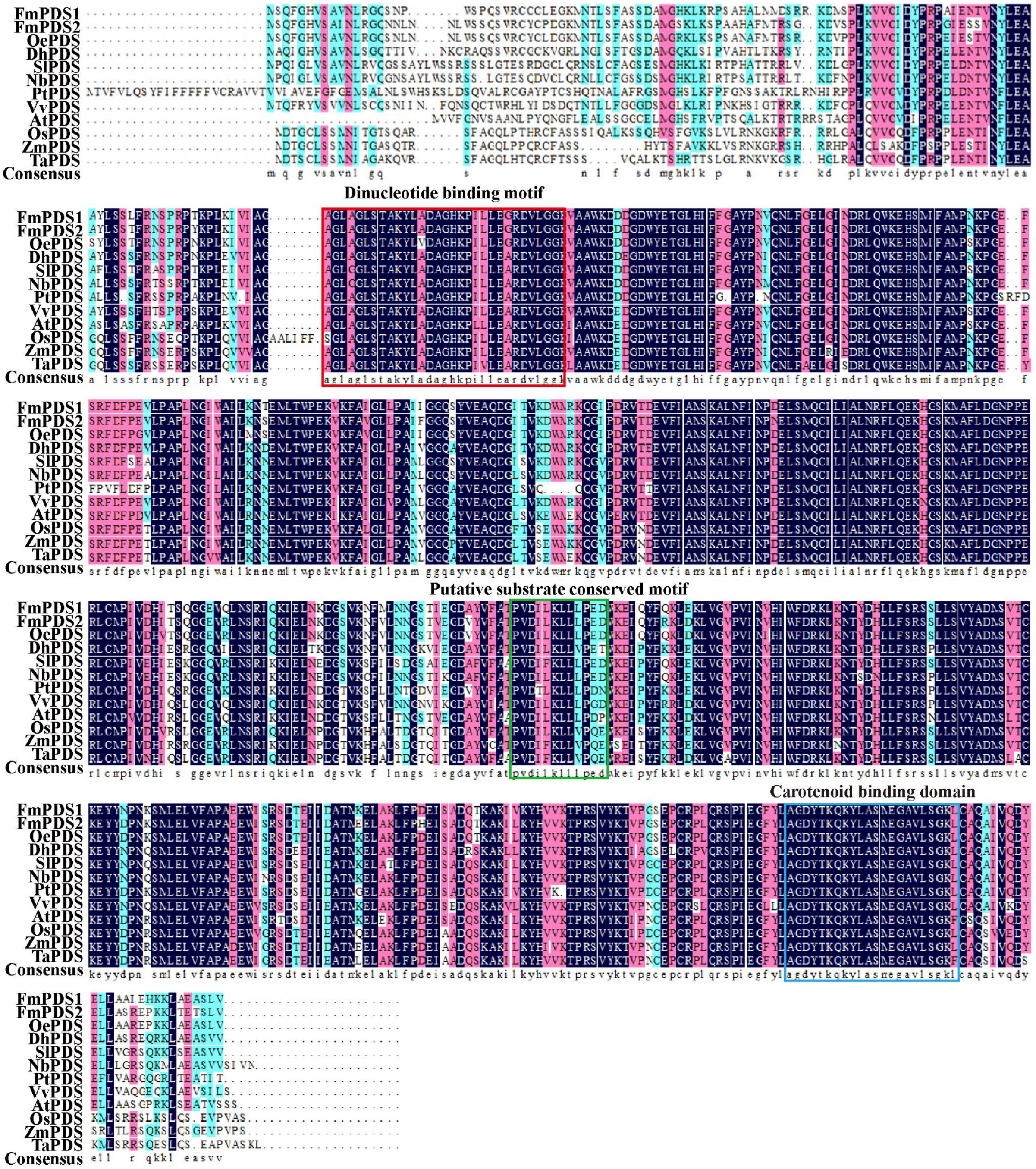

Supplement: Supplementary file 1 — Supplementary data to this article can be found online. [file FR-2025-5-0016-Supplementary.zip › 10.48130_forres-0025-0016-Suppl-FigureS3.pdf]
