# Supplementary material for: Development of an endogenous promoter-driven CRISPR/Cas9 system for genome editing in Fraxinus mandshurica
Source: For Res (Fayettev). 2025 Aug 4;5:e016. doi: 10.48130/forres-0025-0016 (PMC12441911; doi:10.48130/forres-0025-0016)

Fig. S4 Temperature effect on endogenous promoter activity. (a-c) The expression level of *GUS* driven by promoters of *FmU6-6-4* (a), *FmU6-7-4* (b), and *FmECP3* (c) in seedlings of *F. mandshurica* under different temperature (22℃, 28℃, 32℃, 37℃) treatments. Different letters indicated significant differences (P<0.05).

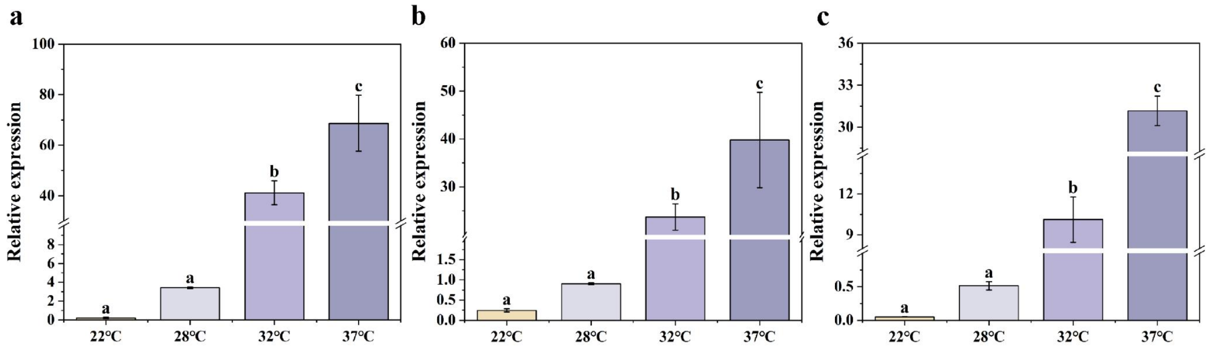

Supplement: Supplementary file 1 — Supplementary data to this article can be found online. [file FR-2025-5-0016-Supplementary.zip › 10.48130_forres-0025-0016-Suppl-FigureS4.pdf]
