# Supplementary material for: Development of an endogenous promoter-driven CRISPR/Cas9 system for genome editing in Fraxinus mandshurica
Source: For Res (Fayettev). 2025 Aug 4;5:e016. doi: 10.48130/forres-0025-0016 (PMC12441911; doi:10.48130/forres-0025-0016)

Fig. S5 The influence of light quality combinations on tissue culture in *F. mandshurica*. G1, white light in the whole stages; red light in germination, white light in elongation and B:R=1:4 (G2), B:R=2:3 (G3) or white light (G4) in rooting; white light in germination and elongation stages, as well as B:R=1:4 (G5) or B:R=2:3 (G6) in rooting; B, blue light; R, red light. Different letters indicated significant differences (P<0.05).

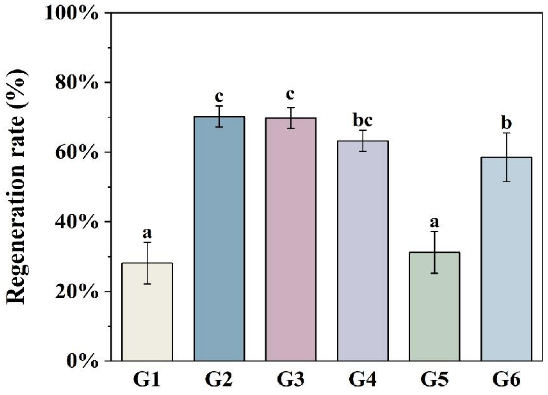

Supplement: Supplementary file 1 — Supplementary data to this article can be found online. [file FR-2025-5-0016-Supplementary.zip › 10.48130_forres-0025-0016-Suppl-FigureS5.pdf]
