# Supplementary material for: Development of an endogenous promoter-driven CRISPR/Cas9 system for genome editing in Fraxinus mandshurica
Source: For Res (Fayettev). 2025 Aug 4;5:e016. doi: 10.48130/forres-0025-0016 (PMC12441911; doi:10.48130/forres-0025-0016)
Supplement: Supplementary file 1 — Supplementary data to this article can be found online. [file FR-2025-5-0016-Supplementary.zip › 10.48130_forres-0025-0016-Suppl-TableS1.pdf]

Table S1: Primer sequences for cloning.

ID	Sequence (5' to 3')
FmU6-3-F	ACCACCGTCGTCTCCTCCA
FmU6-3-R	CATCCAGAAGTTCACCTAAAGCA
FmU6-5-F	AGACAGCAAAGCACCTTGAGAG
FmU6-5-R	TTATTGGTGGAACCCGCC
FmU6-6-F	ACATCAACTCCAACACCGCC
FmU6-6-R	GACGAGAGGAACGACGGAAA
FmU6-7-F	TATACCAACCATTCCTTCTCACTG
FmU6-7-R	TTCTGGACAAAACCCTGGC
FmU6-6-1-F	CCAACCACCAACCGCATGT
FmU6-6-2-F	ACAGTTCGATTGAACTGTGACTTC
FmU6-6-3-F	GGCAATCCATTAGACTTTTGAG
FmU6-6-4-F	GAAGGTGTGGCGAGAAATCTTAT
FmU6-6-1/2/3/4-R	AATTTTATCGGATGTCCCCG
FmU6-7-4-F	TAACATCGTTGGGTAAATG
FmU6-7-4-R	AATTTTATCGGATGTCCCCG
FmECP1-F	TCGTCATCCCAGTTTCTTCA
FmECP1-R	AAGCTCACCCAAGTTAGATTGT
FmECP2-F	TTTGGTTGACGATCATGTGG
FmECP2-R	AAAATTCACCGGGAGACAGA
FmECP3-F	GTTGGGAGAAGGGGTTGAA
FmECP3-R	CAGTTGATGGGTCGTGGAG
FmECP4-F	GGGCCTACTTTATTGAATGGT
FmECP4-R	GGATAGTTTGGGAAATGTGGTT
FmECP5-F	TTGTCACGGTGCCACAAC
FmECP5-R	GCCCAAAGGAAATTCTCATC
FmECP6-F	TGAGCGGTCCGAAATAACC
FmECP6-R	GCCCACAACCAAAAGCAGA
FmECP7-F	CGGAATAAAGCTGAGACGTG
FmECP7-R	AGGGACTCCAACAAGGAAGA
FmECP8-F	TATGTCCTCAAATATCCCCTCT
FmECP8-R	CACCTACCCCTACAATAACTCC
FmECP9-F	TGGACTTGTGGCTTTCGG
FmECP9-R	AGAACGAGGGAATGGGTTTA
FmECP10-F	TGATTTAGCCCCTGTTTGAA
FmECP10-R	CAATTGTTGGTGTCAGTTTGC
FmECP11-F	AGTGTCCGTTGGAATGAATG
FmECP11-R	CCAAGTGATGAAGGGAGATGTA
