# Supplementary material for: Development of an endogenous promoter-driven CRISPR/Cas9 system for genome editing in Fraxinus mandshurica
Source: For Res (Fayettev). 2025 Aug 4;5:e016. doi: 10.48130/forres-0025-0016 (PMC12441911; doi:10.48130/forres-0025-0016)
Supplement: Supplementary file 1 — Supplementary data to this article can be found online. [file FR-2025-5-0016-Supplementary.zip › 10.48130_forres-0025-0016-Suppl-TableS2.pdf]

**Table S2: Primer sequences for vector construction.**

| ID | Sequence (5' to 3') |
|---|---|
| pNC-121-proAtU6-26-F | CAGTGGTCTCTGTCCAGTCCTAAGCTTTCGTTTTCTTCTTTTTAACTT |
| pNC-121-proAtU6-26-R | CGGTCTCAGCAGACCACAAGTCAATCACTACTTCGACTCTAGCTGTATATAAA |
| pNC-121-proFmU6-3-F | CAGTGGTCTCTGTCCAGTCCTACCACCGTCGTCTCCTCCA |
| pNC-121-proFmU6-3-R | CGGTCTCAGCAGACCACAAGTCATCCAGAAGTTCACCTAAAGCA |
| pNC-121-proFmU6-5-F | CAGTGGTCTCTGTCCAGTCCTAGACAGCAAAGCACCTTGAGAG |
| pNC-121-proFmU6-5-R | CGGTCTCAGCAGACCACAAGTTTATTGGTGGAACCCGCC |
| pNC-121-proFmU6-6-F | CAGTGGTCTCTGTCCAGTCCTACATCAACTCCAACACCGCC |
| pNC-121-proFmU6-6-R | CGGTCTCAGCAGACCACAAGTGACGAGAGGAACGACGGAAA |
| pNC-121-proFmU6-7-F | CAGTGGTCTCTGTCCAGTCCTTATACCAACCATTCCTTCTCACTG |
| pNC-121-proFmU6-7-R | CGGTCTCAGCAGACCACAAGTTTCTGGACAAAACCCTGGC |
| pNC-121-proFmU6-6-1-F | CAGTGGTCTCTGTCCAGTCCTCCAACCACCAACCGCATGT |
| pNC-121-proFmU6-6-2-F | CAGTGGTCTCTGTCCAGTCCTACAGTTCGATTGAACTGTGACTTC |
| pNC-121-proFmU6-6-3-F | CAGTGGTCTCTGTCCAGTCCTGGCAATCCATTAGACTTTTGAG |
| pNC-121-proFmU6-6-4-F | CAGTGGTCTCTGTCCAGTCCTGAAGGTGTGGCGAGAAATCTTAT |
| pNC-121-proFmU6-6-1/2/3/4-R | CGGTCTCAGCAGACCACAAGTAATTTTATCGGATGTCCCCG |
| pNC-121-proFmU6-7-4-F | CAGTGGTCTCTGTCCAGTCCTTAACATCGTTGGGTAAATG |
| pNC-121-proFmU6-7-4-R | CGGTCTCAGCAGACCACAAGTAATTTTATCGGATGTCCCCG |
| pNC-121-35S(Enhanced)-F | CAGTGGTCTCTGTCCAGTCCTATGAGACTTTTCAACAAAGGG |
| pNC-121-35S(Enhanced)-R | CGGTCTCAGCAGACCACAAGTTCAGCGTGTCCTCTCCAA |
| pNC-121-proFmECP1-F | CAGTGGTCTCTGTCCAGTCCTTCGTCATCCCAGTTTCTTCA |
| pNC-121-proFmECP1-R | CGGTCTCAGCAGACCACAAGTAAGCTCACCCAAGTTAGATTGT |
| pNC-121-proFmECP2-F | CAGTGGTCTCTGTCCAGTCCTTTTGGTTGACGATCATGTGG |
| pNC-121-proFmECP2-R | CGGTCTCAGCAGACCACAAGTAAAATTCACCGGGAGACAGA |
| pNC-121-proFmECP3-F | CAGTGGTCTCTGTCCAGTCCTGTTGGGAGAAGGGGTTGAA |
| pNC-121-proFmECP3-R | CGGTCTCAGCAGACCACAAGTCAGTTGATGGGTCGTGGAG |
| pNC-121-proFmECP4-F | CAGTGGTCTCTGTCCAGTCCTGGGCCTACTTTATTGAATGGT |
| pNC-121-proFmECP4-R | CGGTCTCAGCAGACCACAAGTGGATAGTTTGGGAAATGTGGTT |

| | |
|---|---|
| pNC-121-proFmECP5-F | CAGTGGTCTCTGTCCAGTCCTTTGTCACGGTGCCACAAC |
| pNC-121-proFmECP5-R | CGGTCTCAGCAGACCACAAGTGCCCAAAGGAAATTCTCATC |
| pNC-121-proFmECP6-F | CAGTGGTCTCTGTCCAGTCCTTGAGCGGTCCGAAATAACC |
| pNC-121-proFmECP6-R | CGGTCTCAGCAGACCACAAGTGCCCACAACCAAAAGCAGA |
| pNC-121-proFmECP7-F | CAGTGGTCTCTGTCCAGTCCTCGGAATAAAGCTGAGACGTG |
| pNC-121-proFmECP7-R | CGGTCTCAGCAGACCACAAGTAGGGACTCCAACAAGGAAGA |
| pNC-121-proFmECP8-F | CAGTGGTCTCTGTCCAGTCCTTATGTCCTCAAATATCCCCTCT |
| pNC-121-proFmECP8-R | CGGTCTCAGCAGACCACAAGTCACCTACCCCTACAATAACTCC |
| pNC-121-proFmECP9-F | CAGTGGTCTCTGTCCAGTCCTTGGACTTGTGGCTTTCGG |
| pNC-121-proFmECP9-R | CGGTCTCAGCAGACCACAAGTAGAACGAGGGAATGGGTTTA |
| pNC-121-proFmECP10-F | CAGTGGTCTCTGTCCAGTCCTTGATTTAGCCCCTGTTTGAA |
| pNC-121-proFmECP10-R | CGGTCTCAGCAGACCACAAGTCAATTGTTGGTGTCAGTTTGC |
| pNC-121-proFmECP11-F | CAGTGGTCTCTGTCCAGTCCTAGTGTCCGTTGGAATGAATG |
| pNC-121-proFmECP11-R | CGGTCTCAGCAGACCACAAGTCCAAGTGATGAAGGGAGATGTA |
| pEgU7E3-FmU6-6-4-F | CAATTAGGGCCCCGCTAGCAAGCTTGAAGGTGTGGCGAGAAATCTTAT |
| pEgU7E3-FmU6-6-4-R | GCTATTTCTAGCTCTAAAACTGAGACCCTCTCTCGGTCTCCAATTTTATCGGATGTCCCCG |
| pEgU7E3-FmU6-7-4-F | CAATTAGGGCCCCGCTAGCAAGCTTTAACATCGTTGGGTAAATG |
| pEgU7E3-FmU6-7-4-R | GCTATTTCTAGCTCTAAAACTGAGACCCTCTCTCGGTCTCCAAGTCATCTAGCTATCACAT |
| pEgU7E3-FmECP3-F | ATTTGAATTCCGCGGTGGCGCGCCGTTGGGAGAAGGGGTTGAA |
| pEgU7E3-FmECP3-R | GGTAATTGTTGTAAAAATACTCTAGACAGTTGATGGGTCGTGGAG |
| pEgU7E3-FmPDS-sgRNA4-F | ACTTGAAGGAAGGGATGTCCTGGG |
| pEgU7E3-FmPDS-sgRNA4-R | AAACCCCAGGACATCCCTTCCTTC |
| pEgU7E3-FmPDS-sgRNA6-F | ACTTGACCATATTACTTCACAAGG |
| pEgU7E3-FmPDS-sgRNA6-R | AAACCCTTGTGAAGTAATATGGTC |
