# Supplementary material for: Development of an endogenous promoter-driven CRISPR/Cas9 system for genome editing in Fraxinus mandshurica
Source: For Res (Fayettev). 2025 Aug 4;5:e016. doi: 10.48130/forres-0025-0016 (PMC12441911; doi:10.48130/forres-0025-0016)
Supplement: Supplementary file 1 — Supplementary data to this article can be found online. [file FR-2025-5-0016-Supplementary.zip › 10.48130_forres-0025-0016-Suppl-TableS3.pdf]

Table S3: Primer sequences for qRT-PCR.

ID	Sequence (5' to 3')
FmActin-F	AGGACGCTGCCAACAACTTT
FmActin-R	TTGAGGGGAAGGGTAAATAGTG
DL-GUS-F	CGATGCGGTCACTCATTA
DL-GUS-R	CCAGTTCAGTTCGTTGTTC
DL-sgRNA4-F	GGTTTGGCTGGTTTGTCTACT
DL-sgRNA4-R	GGTTCTGCACATTTGGGTAA
DL-Cas9-F	AGGTTGTGGATGAGTTGGTG
DL-Cas9-R	TCTCAACAGGGTGCTCTTTG
