# Supplementary material for: Development of an endogenous promoter-driven CRISPR/Cas9 system for genome editing in Fraxinus mandshurica
Source: For Res (Fayettev). 2025 Aug 4;5:e016. doi: 10.48130/forres-0025-0016 (PMC12441911; doi:10.48130/forres-0025-0016)
Supplement: Supplementary file 1 — Supplementary data to this article can be found online. [file FR-2025-5-0016-Supplementary.zip › 10.48130_forres-0025-0016-Suppl-TableS4.pdf]

Table S4: Primer sequences for gRNA efficiency detection.

ID	Sequence (5' to 3')
T7-sgRNA4	TTAATACGACTCACTATAGGGGAAGGAAGGGATGTCCTGGGGTTTTAGAGCTAGAAATA
T7-sgRNA6	TTAATACGACTCACTATAGGGGACCATATTACTTCACAAGGGTTTTAGAGCTAGAAATA
FmPDS1-sgRNA4-F	AGTTAGCTGTTAGTGCTTTGCG
FmPDS1-sgRNA4-R	CTTGCTTTCTCATCCAGTCTTT
FmPDS1-sgRNA6-F	GGCACTGAACTTCATTAACCC
FmPDS1-sgRNA6-R	ATGGCAACTCACAGACAACACT
FmPDS2-sgRNA4-F	TCCCCTTTGAAGGTATGGC
FmPDS2-sgRNA4-R	TTCCCCTGGCTTGTTTGG
FmPDS2-sgRNA6-F	GAAGAAGCAAGACCAAATCG
FmPDS2-sgRNA6-R	CAGAACAGGGGTGGAGACA
DL-dna-FmPDS1-sgRNA4-F	AAGTACTTGGCAGATGCAGG
DL-dna-FmPDS1-sgRNA4-R	CAGTCTCATACCAGTCTCCATC
DL-dna-FmPDS1-sgRNA6-F	GAAGCATGGTTCAAAGATGGC
DL-dna-FmPDS1-sgRNA6-R	CATCTCCTTCAATCGTACTCCC
DL-dna-FmPDS2-sgRNA4-F	GTTTGGCTGGTTTGTCTACTG
DL-dna-FmPDS2-sgRNA4-R	CACAACGAGGGACAAATTCAG
DL-dna-FmPDS2-sgRNA6-F	CACCAGAAAGACTTTGCATGC
DL-dna-FmPDS2-sgRNA6-R	ATCTCCTTCTACCGTACTCCC
