# Supplementary material for: Development of an endogenous promoter-driven CRISPR/Cas9 system for genome editing in Fraxinus mandshurica
Source: For Res (Fayettev). 2025 Aug 4;5:e016. doi: 10.48130/forres-0025-0016 (PMC12441911; doi:10.48130/forres-0025-0016)
Supplement: Supplementary file 1 — Supplementary data to this article can be found online. [file FR-2025-5-0016-Supplementary.zip › 10.48130_forres-0025-0016-Suppl-TableS5.pdf]

Table S5: Primer sequences for mutation detection.

ID	Sequence (5' to 3')
YZ-F	CGGGCCTCTTCGCTATTA
YZ-R	AAACCCCAGGACATCCCTTCCTTC
YZ-OTS1-F	TGCCAATGGATACCACAAAG
YZ-OTS1-R	GTGCTATTCATACTGTCCAAAGAC'
YZ-OTS2-F	AGCCGAAGGGCAATCTT
YZ-OTS2-R	CCAGCTCGTGTTTCCAGTG
YZ-OTS3-F	CGTAAATAAATGGGACGGAG
YZ-OTS3-R	GAATTGTGGGCATTGAGATG
YZ-OTS4-F	GTCTCCATCATCATCTTTCCAT
YZ-OTS4-R	TTGGCTGGTTTGTCTACTGC
YZ-OTS5-F	GCAATAATTGGTCAACGTCTTC
YZ-OTS5-R	CATCTTGTTACTGGTTTCCTGTCT
YZ-Cas9-F	AGGTTGTGGATGAGTTGGTG
YZ-Cas9-R	TCTCAACAGGGTGCTCTTTG
HITOM-FmPDS1-F	GAGTACGGTGTGCAGGTTTGGCTGGTTTGTCTAC
HITOM-FmPDS1-R	GGATGCTGGATGGTAGGTTCTGCACATTTGGGT
HITOM-FmPDS2-F	GAGTACGGTGTGCTTAGTCAAAGATTTATTTAATG
HITOM-FmPDS2-R	GGATGCTGGATGGTTTGTATTATGCATCAAATCACTTA
