# Supplementary material for: Development of an endogenous promoter-driven CRISPR/Cas9 system for genome editing in Fraxinus mandshurica
Source: For Res (Fayettev). 2025 Aug 4;5:e016. doi: 10.48130/forres-0025-0016 (PMC12441911; doi:10.48130/forres-0025-0016)
Supplement: Supplementary file 1 — Supplementary data to this article can be found online. [file FR-2025-5-0016-Supplementary.zip › 10.48130_forres-0025-0016-Suppl-TableS6.pdf]

Table S6: Summary of the edited genes and phenotypes of the sequenced transgenic plants.

		Edited or not					
		**#3**	**#9**	**#11**	**#12**	**#13**	**#14**
Gene name	*FmPDS1*	Y	N	N	Y	N	Y
	FmPDS2	N	N	N	N	N	N
		-2			-2		-21, +2
Phenotype		Pale green	Green	Green	Pale green	Green	Pale green

*Y and N represents the gene is edited or not.
