# Supplementary material for: Development of an endogenous promoter-driven CRISPR/Cas9 system for genome editing in Fraxinus mandshurica
Source: For Res (Fayettev). 2025 Aug 4;5:e016. doi: 10.48130/forres-0025-0016 (PMC12441911; doi:10.48130/forres-0025-0016)
Supplement: Supplementary file 1 — Supplementary data to this article can be found online. [file FR-2025-5-0016-Supplementary.zip › 10.48130_forres-0025-0016-Suppl-TableS7.pdf]

Table S7: Summary of off-target site sequencing results for mutations (#1 and #3).

Off-target site	WT	Number of matching bases	#1	#3
FmPDS1/2 genotype at sgRNA4 site	GAAGGAAGGGATGTCCTGGG	20	+1	G-T
Off-target site 1 (Chr09G)	AGAGGAAGGGATGTCCTTTC	15	no change	no change
Off-target site 2 (Chr04G)	GAAGGAAGGGATGTTAAGGT	16	no change	no change
Off-target site 3 (Chr22G)	GAAGGAAGGGATGTAATTTT	15	no change	no change
Off-target site 4 (Chr15G)	CAAGGAAGGGATGTCTATTA	14	no change	no change
Off-target site 5 (Chr10G)	GAAGGAAGGGATGTGTTAAGAA	15	no change	no change

*Note: Black nucleotides are homologous between FmPDS1/2 sgRNA 4 site and the off-target site.
